# When Barriers Break: Tight Junction Regulation and Dynamic Alterations of Barrier Integrity in Neurological Injury

**DOI:** 10.3390/cells15030232

**Published:** 2026-01-26

**Authors:** Kayli N. Colpitts, James W. Grau

**Affiliations:** 1 Institute for Neuroscience, Texas A&M University, College Station, TX 77840, USA; j-grau@tamu.edu; 2 Psychological and Brain Sciences, Texas A&M University, College Station, TX 77840, USA

**Keywords:** blood-brain barrier, blood-spinal cord barrier, neural injury, neuroinflammation, barrier disruption, tight junctions, tight junction proteins

## Abstract

The blood–brain barrier and blood–spinal cord barrier (BBB/BSCB) are essential protective components for the healthy functioning of the central nervous system (CNS). While these barriers protect the CNS from peripheral factors, such as immune cells and blood products, they can become disrupted in pathological conditions and injury. The neurovascular unit (NVU) is composed of endothelial cells (ECs), pericytes, astrocytes, microglia, and neurons, all of which contribute to proper function and the maintenance of the BBB/BSCB. Tight junctions (TJs) unite cellular components and are modulated by both intrinsic and extrinsic factors. Systemic processes, such as pain (nociceptive activity), inflammation, and blood hemostasis, can impact BBB/BSCB function, often leading to a disrupted barrier and increased peripheral infiltration. This, in turn, can increase neuroinflammation and drive microglia activation, progressive hemorrhagic necrosis (PHN), and matrix metalloproteinase (MMP) activity. Targeting these processes and mitigating the deleterious effects of BBB/BSCB breakdown represents a key therapeutic target after neural injury and other pathological conditions.

## 1. Introduction

The blood–brain barrier (BBB) and blood–spinal cord barrier (BSCB) are critical membranes that separate the central nervous system (CNS) from the vascular system and the periphery [1]. These barriers maintain homeostasis by selectively preventing the entry of harmful substances while dynamically regulating the influx of nutrients and metabolites necessary to fuel vital cellular processes. This balance is dependent upon the barriers’ ability to sense and respond to internal and external cues and regulate the passage of beneficial molecules while remaining impermeable to cytotoxic compounds. In the setting of various disease and injury states, the BBB and BSCB may fail to protect the CNS from harmful substances, highlighting the need to identify new therapeutic treatments. As reviewed here, some molecules may reduce barrier permeability and thereby act as protective and stabilizing factors. Conversely, other substances may increase barrier permeability and promote disruption.

Neural injuries, such as traumatic spinal cord injury (SCI) or traumatic brain injury (TBI), are associated with the breakdown of the BSCB and BBB, respectively [2,3,4]. In neural injury, there are two stages that have been characterized: the primary mechanical injury that is a direct result of the insult and the secondary tissue loss that follows [5,6]. The disruption due to physical insult results in the influx of immune cells and other peripheral factors that fuel secondary injury. This infiltration triggers downstream effects that amplify the neuroimmune response, disrupting normal signaling and homeostasis within the CNS. In addition to neural injuries, other reviews have noted a relationship between barrier disruption and neuropathologies such as epilepsy [7,8], ischemic stroke [9], and Alzheimer’s disease [10]. Alterations in barrier permeability are also evident in systemic models, such as sepsis [11,12] and hypertension [13]. The current review focuses on BBB/BSCB breakdown in the context of neural injury and pathologies known to be comorbid with neural injuries.

The functional properties of the BBB/BSCB are tied to a variety of cell types: endothelial cells (ECs), pericytes, glial cells, and neurons [1]. The ECs are the primary physical barrier and rely on specific protein complexes that mediate the cell-to-cell junctions, adhesion, and signaling to maintain barrier integrity. Two protein complexes, tight junctions (TJs) and adherens junctions, play a pivotal role. Although both are involved in BBB function, the restrictive properties of the ECs are typically linked to TJs, while adherens junctions are characterized as playing a supportive role, contributing to the formation and maintenance of TJs themselves [14,15].

We explore the role of TJs in the maintenance of barrier integrity, the impact of extrinsic and intrinsic factors that impact TJ function, and how this affects BBB integrity, with a particular focus on clinical outcomes and therapeutic targets. We begin with an overview of the cellular components of the BBB (2), the protein composition of TJs (3), and how these systems develop and function in the absence of pathology (4). We then consider extrinsic factors that can impact BBB integrity (5), detailing the effects of pain, inflammation, and hemostatic processes (coagulopathy and fibrinolysis). Next, we consider intrinsic factors (6), such as the expression of proinflammatory cytokines, phagocytosis, and progressive hemorrhagic necrosis. The last section highlights the implications of this work, reviewing current clinical and preclinical research and how these processes contribute to tissue loss after neural injury (7).

## 2. The Blood–Brain Barrier: Cellular Components and Function

The first three sections of the current review provide an overview of the cellular composition of the BBB, the proteins that form TJs, and BBB function in the absence of pathology or injury. The material provides the foundation for understanding later sections, which discuss the factors that contribute to barrier dysfunction. Readers familiar with BBB composition, TJPs, and BBB function may wish to skip ahead to Section 5.

Here, and in the remainder of this paper, we will generally refer to the barrier as the BBB, a nomenclature that recognizes the commonalities between the BBB and BSCB [16]. At the same time, it is important to note the key difference in barrier function. The BSCB is more permeable than the BBB to tracers such as radiolabeled mannitol and carboxyl-inulin [17]. A similar trend for increased BSCB permeability has been noted with cytokines such as interferon gamma (IFN-γ) and tumor necrosis factor α (TNF-α) [18]. These differences in permeability have been related to tight junction proteins (TJPs) [19], and for this reason, our discussion of how TJPs contribute to barrier permeability is relevant to understanding how the BBB and BSCB differ. Where appropriate, we will note differences in BBB and BSCB function, recognizing that further work is needed to address mechanistic differences.

### 2.1. Cellular Composition of the BBB

**Figure 1:**
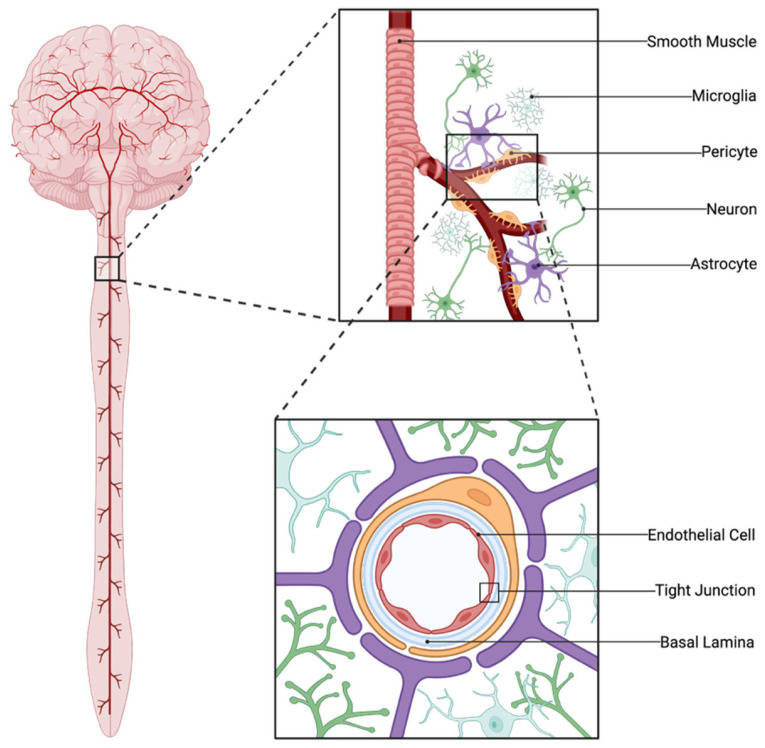
Key components of the NVU. Vessels throughout the brain and spinal cord are surrounded by the NVU, which is composed of ECs, pericytes, astrocytes, microglia, and neurons. The NVU dynamically responds to stimuli and modulates blood flow as well as BBB/BSCB function. Note: Adapted from “Blood-brain barrier dysfunction in normal aging and neurodegeneration: Mechanisms, impact, and treatments.” by A. V. Andjelkovic et al., 2023 [23], *Stroke*, page 663. Created with Biorender.com.

The BBB is composed of ECs lining blood vessels, the capillary basement membrane, including pericytes and glial cells [1,20,21]. The neurovascular unit (NVU) refers to the structural components and interactions between neuronal, glial, and vascular cells. These cells work in concert to modulate blood flow, in addition to sustaining the maintenance and permeability of the BBB [22]. The primary function of the NVU is neurovascular coupling, which ensures that activated brain regions receive adequate blood flow to supply nutrients to the area. The following sections will review the cell types that form the NVU, with a particular focus on their role in the BBB and the methods used to assess barrier function. Key components of the NVU are illustrated in Figure 1.

#### 2.1.1. Endothelial Cells (ECs)

ECs form the lumen of blood vessels and play a critical role in maintaining impermeability. Brain microvascular ECs have been shown to have enriched expression of TJPs such as claudin-5 (CLDN-5) and occludin (OCLN) [24,25]. ECs are able to alter the barrier through crosstalk with other cells of the NVU, mediated in part by the release and binding of cytokines and chemokines (reviewed in [26,27,28,29]). Activation of ECs leads to increased production of cytokines, greater permeability, and the expression of adhesion molecules. Expression of adhesion molecules, such as intercellular adhesion molecule-1 (ICAM-1) and vascular cell adhesion molecule-1 (VCAM-1), serves as a signal that marks the site of injury, attracts circulating immune cells, and enables leukocyte transmigration [30]. This results in increased immune cell infiltration, further EC activation, and apoptosis. Infiltration of immune cells, such as neutrophils and T cells, is harmful to the CNS and initiates a vicious cycle leading to continued barrier disruption. Neutrophils lead to the release of matrix metalloproteinases, neutrophil extracellular traps, and other proinflammatory mediators [31,32]. These factors promote proteolytic cleavage of TJPs and TJ internalization [31,32]. Others have shown a similar disruption wherein T cells result in significant increases in barrier permeability via the disruption of TJP organization [33]. Additionally, cerebral ischemia has been shown to increase expression of ICAM-1, VCAM-1, and the neutrophil chemoattractant C-X-C Motif Chemokine Ligand 1 (CXCL1). This, in turn, increased neutrophil transendothelial migration, which is known to occur following the expression of cell adhesion molecules [30]. The position of ECs as the primary contact between the CNS and vasculature provides a potential therapeutic target.

#### 2.1.2. Pericytes

Pericytes on capillaries and within the CNS contribute to the maintenance/functioning of the BBB and the regulation of blood flow (for reviews, see [34,35,36,37]). Identified by their expression of the platelet-derived growth factor receptor β (PGDFB) and chondroitin sulfate proteoglycan 4 (CSPG4, commonly called NG2), confirmed with single-cell transcriptomics [24,25], pericytes vary in morphology according to their location within capillary beds [35,36,37]. Pericytes are thought to have physiological functions similar to arteriole smooth muscle cells and, depending on the type, may regulate blood flow abruptly or more slowly [36,37,38,39]. Because changes in blood flow are observed in capillaries prior to arterioles, some have suggested that capillaries (and thereby the contracting pericytes) are the primary regulators of blood flow within the CNS [39]. These pericytes have a diverse expression of ion channels, almost half of which are ATP-sensitive potassium channels (K_ATP_) that allow the pericyte to hyperpolarize in response to changes in the microenvironment [37,40]. This hyperpolarization is then transmitted to ECs via gap junctions and eventually relayed back to penetrating arterioles to modulate blood flow. This metabolic sensing and dynamic pericyte response is crucial to local blood flow control.

While implicated in blood flow, hippocampal pericytes have also been linked to the formation of long-term memory [41]. Changes in insulin-like growth factor 2 (IGF2) are driven by increases in pericytes, and this process is dependent on neuronal activity. Pericyte-specific inducible knock out (KO) of IGF2 impaired memory of novel object location and contextual fear [41].

In addition to roles in blood flow and memory formation, pericytes can alter barrier function, as their depletion results in increased permeability [35,38,42,43]. Pericytes reduce the expression of factors contributing to vascular permeability, such as angiopoietin-2 (ANGPT-2), matrix metalloproteinase-9 (MMP-9), and cellular adhesion molecules that lead to immune cell infiltration. Clinically, pericyte loss in the brain has been associated with increased barrier permeability in pathological conditions such as diabetes, stroke, and TBI. Pericyte loss can also occur in other tissues, such as in the lungs following stroke and in the kidneys of individuals with diabetes [35].

Pericytes also play a role in BBB development [38,42,43]. They are implicated in the maturation and organization of the TJs. Because pericytes are found around capillaries throughout the body and are implicated in angiogenesis, it has been difficult to produce viable KO models. Disruption of PDGFRB signaling has been used to drive pericyte Kos, but is perinatally lethal. Because of this, other genetic models that yield varying levels of pericyte deficiency have been developed, yielding lower pericyte counts at birth [38]. Interestingly, deficiency often results in a progressive, age-dependent loss of pericytes throughout development and into adulthood. This age-dependent pericyte loss is accompanied by other physiological alterations, including shorter capillary length, reduced cerebral perfusion and blood flow, increased barrier permeability to endogenous plasma proteins and exogenous dextran tracers, and a reduction in zonula occluden-1 (ZO-1) and occludin (OCLN) protein expression [38].

#### 2.1.3. Astrocytes

Astrocytes are the most abundant cell type in the CNS and contribute to numerous homeostatic processes (reviews regarding astrocyte functioning in health and disease: [44,45,46]). These cells participate in neurovascular coupling, synaptic regulation, and maintenance of energy stores [46]. While they are not implicated in the early development of the BBB, which is already formed by the time astrocytes develop, they are involved in the maturation and maintenance of the barrier [44,45,46]. Astrocytes are heterogeneous in nature and exhibit dynamic cellular adaptations in response to injury or inflammation, evident from single-cell RNA sequencing [24,25,47].

Astrocytes secrete a number of factors that can have either harmful or protective effects on BBB/BSCB permeability [44]. Permeability is increased through the secretion of astrocyte-derived factors that lead to the degradation of TJPs and increased leukocyte infiltration via upregulation of adhesion molecules. Examples include vascular endothelial growth factor (VEGF), matrix metalloproteinases (MMPs), nitric oxide (NO), glutamate (excessive levels), and endothelins. Some of these factors, such as VEGF, NO, and glutamate, are important in healthy functioning as well. VEGF is typically involved in EC proliferation and angiogenesis, but in the setting of an injury, it can increase permeability by down-regulating TJP and promoting peripheral immune cell transmigration. Glutamate-induced NMDA receptor activation can elicit vasodilation, which requires NOS-3, and at excessive levels, it increases permeability. While the mechanisms that underlie these effects are still debated, some have linked these effects to a reduction in OCLN levels [44].

Astrocytes also release factors that decrease barrier permeability. Examples include angiopoietin-1 (ANGPT-1), sonic hedgehog (SHH), glial-derived neurotrophic factor (GDNF), retinoic acid (RA), insulin-like growth factor-1 (IGF-1), and apolipoprotein E (APOE). A number of these factors are involved in development and/or angiogenesis as well. For example, SHH is a well-known glycoprotein implicated in the patterning of the body that also acts to prevent EC apoptosis and increase the expression of TJPs [44].

Astrocytes also play a key role in waste removal from the extracellular space [45]. The cells internalize plasminogen activator and plasmin, as well as facilitate the breakdown of fibrinogen and maturation of brain-derived neurotrophic factor (BDNF) [48]. Astrocytes contribute to the removal of excessive GABA, glycine, glutamate, and potassium, and disruption of this process has adverse physiological consequences [45]. Evidence suggests that exposing reactive astrocytes to blood products (e.g., albumin) interferes with the clearance of glutamate and potassium. Accumulation of glutamate can result in excitotoxicity as well as epileptic seizures [45,49,50].

#### 2.1.4. Microglia

Microglia are resident immune cells of the nervous system that work to survey and respond to damage in the CNS (for an in-depth review of microglia in neuropathologies, see [51,52]). In their surveillance role, these cells exhibit a resting state. When damage occurs, microglia are able to react to the injury within minutes and shift to an activated state. When activated, microglia release proinflammatory cytokines, such as TNF-α and interleukin-1β (IL-1β) [51,52], and produce reactive oxygen species (ROS). Microglia can release ROS in a self-perpetuating cycle, wherein the presence of ROS activates microglia, fueling additional ROS production. Microglia activation can be blocked using minocycline, and ROS production can be reduced using diphenyleneiodonium to inhibit NADPH oxidase.

Activated microglia also participate in the phagocytosis of debris. This phagocytic phenotype contributes to difficulty in distinguishing activated microglia from monocyte-derived macrophages. Single-cell transcriptomics is a powerful tool that has been used to differentiate microglia and macrophages and characterize subsets of microglial populations in homeostatic and pathological conditions [24,25,53]. Both ROS production and phagocytosis by microglia have been associated with disruption of the BBB [51,52]. Microglia recognize damage to the CNS and help instigate a neuroimmune response. The neuroimmune response of microglia is implicated in changes in BBB/BSCB function and should be considered when assessing disruption in pathological settings.

#### 2.1.5. Neurons

While neuronal activity is known to regulate hemodynamic changes within the CNS, neurons also modulate the formation and maintenance of the BBB/BSCB [54]. Neuronal cues cause ECs to display the CNS barrier phenotype (reviewed in [52,55,56,57]). Additionally, ECs express glutamate receptors and respond to the release of this neurotransmitter [54]. While essential for neural communication, excessive glutamate release has been shown to increase barrier permeability, which can be blocked by pretreatment with a NMDA receptor antagonist [58]. High levels of glutamate are also linked to epilepsy, a pathological condition associated with BBB disruption [7,8,49,50].

### 2.2. Assessment of BBB/BSCB Permeability

A number of techniques have been developed to assess BBB permeability in vitro and in vivo. Transendothelial electrical resistance (TEER) can be measured in vitro or ex vivo [59,60]. TJs between ECs normally restrict the movement of ions, maintaining an electrical resistance across the EC layer. As a direct measure of paracellular transport, high or increased TEER values are indicative of reduced barrier permeability [59,60].

Barrier function is commonly assessed in vivo by evaluating the ability of molecules to infiltrate the parenchyma [34,59,60,61,62,63]. Injectable compounds come in a variety of sizes that do not penetrate an intact BBB/BSCB. Commonly used compounds include Evan’s blue, dextrans, horseradish peroxidase, and radio-tagged sucrose [60]. In addition to exogenous tracers, extravasation of endogenous blood components such as IgG and fibrinogen can be used [62,63]. Others have evaluated alterations in permeability using molecules known to be dependent on active transport. For example, transferrin has been used to assess receptor-mediated transport, while albumin can be used to monitor caveolar transcytosis [59,61]. Furthermore, in vivo 2-photon microscopy [64] and endomicroscopy (Miniscope) [65] can be paired with the use of fluorescent tracers to assess real-time changes in barrier permeability and vascular morphology.

Many of the procedures used to assess permeability are lethal, making them impossible to utilize in a clinical setting and costly in animal studies. Non-lethal techniques include dynamic contrast-enhanced magnetic resonance imaging (DCE-MRI), positron emission tomography (PET), and single-photon emission computed tomography (SPECT) [34,62,66]. Dynamic contrast-enhanced near-infrared spectroscopy (DCE-NIRS) is a clinical imaging technique used for the assessment of cerebral blood flow, and preclinical work suggests it could provide a minimally invasive method to assess BBB permeability [67].

**Figure 2:**
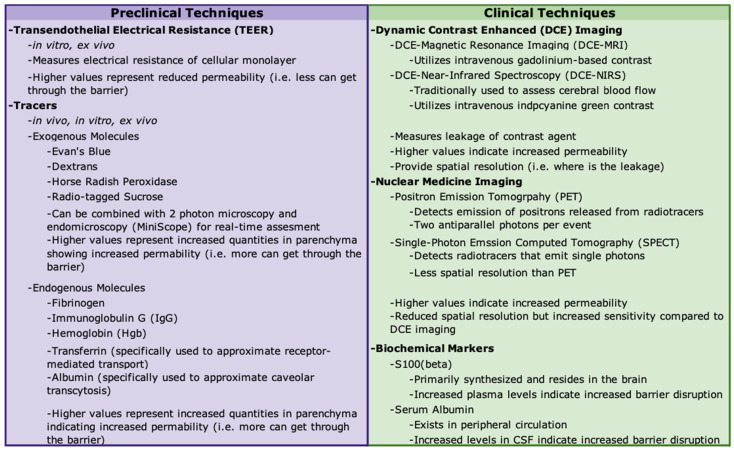
Techniques to assess barrier permeability. Because some preclinical techniques are invasive or involve the application of a harmful agent, they cannot be applied in a clinical setting. Created with Biorender.com.

Current work is seeking to identify additional biomarkers in blood and cerebrospinal fluid (CSF) that can be used to assess barrier disruption non-invasively [34]. The albumin CSF-to-serum ratio can be used to measure the presence of a blood product in CSF. Barrier disruption may be inferred through the converse observation, with the presence of CNS-restricted proteins in systemic circulation [34]. For example, increases in serum levels of S-100β were observed in human patients following mannitol-induced disruption of the BBB for chemotherapy [68,69]. A summary of methods used to assess barrier permeability is provided in Figure 2.

## 3. Tight Junction Proteins Connect ECs to Form a Functional BBB

TJs mediate the connection between ECs and play a key role in maintaining the BBB (for focused reviews, see [34,59,60,70,71]). Adherens junctions are also involved in cell–cell connections but are often located more basally in the barriers [15,59,72]. At a molecular level, multiple proteins contribute to TJ function and the dynamic changes needed to maintain a functional barrier [34,60,71]. In addition, TJs are implicated in cellular polarization and the regulation of cell functions [59].

TJPs are found throughout the body on the apical region of endothelial and epithelial barriers [70,71,73]. TJPs have also been reported in myelinated cells and may play a role in the adhesion and radial components of myelin [74]. Detailed analyses have revealed differential expressions of specific TJPs across barrier types and tissues throughout the body [71].

TJs play a primary role in the maintenance of the BBB and BSCB, forming complexes that have overlapping and repeated regions between brain microvascular ECs [60]. Filaments of integral membrane proteins attach to the cytoskeleton of one EC and protrude to associate with the filaments of a neighboring cell [60,71].

### 3.1. Tight Junctions: Protein Composition

**Figure 3:**
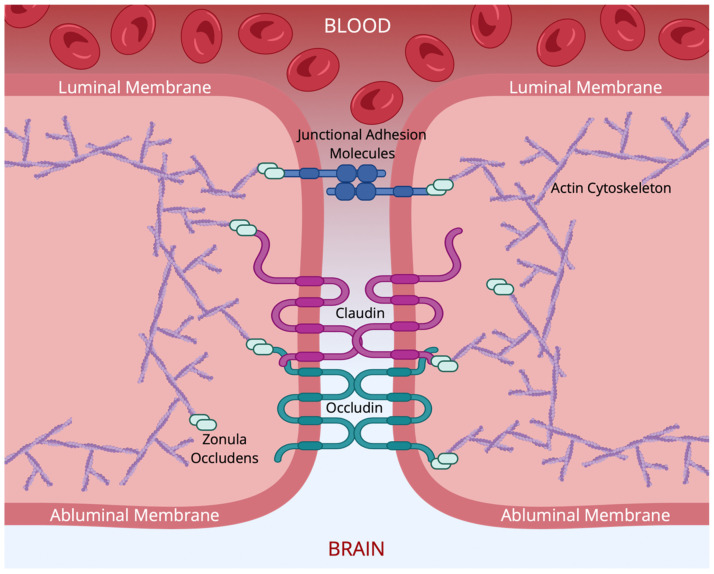
TJP structural organization. TJPs are expressed at connections between neighboring ECs. While CLDN, JAMs, and OCLN are transmembrane proteins, ZOs act as a tether to the actin cytoskeleton. Note: Adapted from “Targeting blood-brain barrier changes during inflammatory pain: An opportunity for optimizing CNS drug delivery” by P. T. Ronaldson and T. P. Davis, 2011 [77], *Therapeutic Delivery*, page 35. Created with Biorender.com.

There are several classes of proteins that are involved in TJs: claudins, tight-junction-associated marvel proteins (TAMPs), zonula occludens (ZOs), and junctional adhesion molecules (JAMs). Claudins and TAMPs are considered the primary contributors to the seal provided by TJs [73]. Intracellular proteins, such as ZOs, and cytoskeleton components (e.g., actin) are implicated in tethering and barrier regulation [73]. JAMs are also involved in the modulation of other TJPs, as well as monocyte migration [75,76]. In the material that follows, we briefly review the major TJPs implicated in the maintenance of the BBB/BSCB. Figure 3 illustrates the structural organization of a TJ.

#### 3.1.1. Claudins

Claudins are a family of 27 proteins that play a key role in maintaining the “tightness” of neighboring ECs [60,78,79]. These proteins are found throughout the body in the endothelial and epithelial barriers and have tissue-specific expression [78]. There are several CLDN isoforms that have been detected within the CNS, including CLDN-1, -3, -5, -11, and -12. Evidence suggests that CLDN-5 is the primary contributor to BBB impermeability and that other isoforms play a small role or contribute to other functions [60,80]. Regulators of CLDN-5 include transforming growth factor β1 (TGF-β1), TNF-α, IL-1β, estrogen, and glutamate [80].

At a structural level, claudins (20–27 kDa) consist of two extracellular loops (ECLs; the first of which is significantly larger), four transmembrane regions, a long C-terminal, and a short N-terminal [59,60,71,80]. The ECLs allow for homo- and hetero-dimerization or oligomerization with claudins on the same (*cis-*) or neighboring (*trans-*) cell membranes. The C-terminus usually includes a PDZ-binding motif that interacts with cytoplasmic proteins, like ZOs. This PDZ-binding motif can also accommodate OCLN and JAM interactions [59,60].

Early studies of claudins linked the proteins to the formation of TJ strands [78,79]. It was proposed and later validated that these strands are formed by CLDN isomers that vary by cell type. The ratio of the isomers expressed determines the tightness of the seal, as certain species form homotypic or heterotypic connections when coupled to specific claudins [79,81,82]. In areas where these bonds are unable to occur, or when occludin or other TJPs are involved, pores may be formed that mediate paracellular ion-selective transport [79,81,82]. This was seen in CLDN-16 deficiency linked to hereditary hypomagnesemia and a lack of magnesium paracellular flux in the renal ascending limb of Henle [79,83].

#### 3.1.2. Tight-Junction-Associated Marvel Proteins (TAMPS)

Occludin (OCLN) was the first protein to be associated with TJs [84]. Despite its association with TJs and its apparent influence on barrier function in vivo and in vitro, its role was not well-understood. OCLN null mice exhibit notable abnormalities, including reduced body size and male infertility. They, however, remain viable and display only mild barrier disruption [84]. This indicates that OCLN contributes to, but is not solely responsible for, maintaining barrier integrity. Following identification of the MARVEL (MAL [Myeloid and Lymphocyte] and related proteins for vesicle trafficking and membrane link) domain in OCLN, tricellulin (MARVELD2) and MARVEL domain-containing protein 3 (MARVELD3) were identified. Further work showed that OCLN is localized to bicellular contacts and MARVELD2 to tricellular contacts [85]. While it is not disputed that MARVELD2 plays a role at the BBB, relatively little is known about its function [34,60,84].

OCLN has four transmembrane domains, a long C-terminal and N-terminal, both within the intracellular region [34,60,85]. The C-terminal has a unique coiled-coil structural motif that allows for binding to cytoplasmic ZO-1. OCLN also has two ECLs, one of which is able to form a disulfide bond under oxidizing conditions. This unique structural component, in conjunction with decreased expression in ischemic/hypoxic conditions, suggests that occludin may act as a redox sensor [85]. Additionally, some groups have linked OCLN to the TJ response elicited by proinflammatory cytokines, such as TNF-α and IFN-γ [85,86].

#### 3.1.3. Junctional Adhesion Molecules (JAMs)

JAMs are members of the immunoglobulin superfamily and are expressed at the BBB/BSCB. Junctional adhesion molecule A (JAM-A) and junctional adhesion molecule B (JAM-B) in particular have been implicated in functional permeability of the CNS barrier [34,76,77]. Notably, JAMs also have the ability to act as adhesion molecules, facilitating monocyte transmigration [76]. More recently, they have been shown to modulate the expression of other TJPs, such as ZO-1 [75]. While JAMs increase monocyte transmigration and modulate other TJPs, their decreased expression is associated with increased barrier permeability [87,88]. Physically, JAMs are a 40 kDa structure with a single transmembrane domain, two V-type immunoglobulin-like loops, two N-glycosylation sites, and an intracellular C-terminal [34,76].

#### 3.1.4. Zonula Occludens (ZOs)

ZOs (130–230 kDa) belong to the membrane-associated guanylate kinases (MAGUK) family and, unlike other TJPs, are intracellular, as opposed to membrane-bound proteins. ZOs generally share the same structure, including a PDZ binding domain and a unique C-terminus that distinguishes isoforms [60]. ZO-1, -2, and -3 are found at the BBB and play an important role in tethering membrane-bound TJPs to the cytoskeleton (primarily ZO-1) [59,60,70]. Despite their high similarity, experimental data suggests that the varying ZO isoforms have distinct roles. Animals deficient in ZO-2 are embryonically lethal, while those deficient in ZO-3 are not [89]. Others have suggested that ZO-1 mediates barrier formation [59,72].

### 3.2. Assessment of TJPs

TJPs can be assessed in a number of ways. Immunofluorescence and immunohistochemical techniques can reveal the organization and localization of the TJPs. In normal physiological conditions, TJs are expressed along the vasculature throughout the CNS in long, strand-like segments, colocalized with various TJPs. In pathological conditions, expression becomes more punctate and discontinuous with lower levels of colocalization. Interestingly, a number of groups have reported changes in the expression of TJPs that do not align linearly with alterations in barrier permeability. In some cases, no TJP changes were observed with barrier disruption. In other cases, limiting degradation of TJPs or increasing their trafficking to the membrane has been shown to rescue barrier function after insult [68,69]. Without assessing localization or the interaction of different TJPs, compensatory mechanisms may overshadow changes observed in overall expression.

Transmission electron microscopy (TEM) can be utilized to visualize the TJs between ECs [59]. The length and width of the “kissing points” where two ECs meet can be quantified. Length (or depth) refers to the extent to which the TJ is maintained across the vertical apical membrane and is negatively correlated with barrier permeability. TJ width refers to the paracellular gap. Under pathological conditions in which the barrier is compromised, kissing points between ECs are often shorter in length but wider. Consequently, both TJ length and width must be measured to assess barrier function [59,85].

Western blotting and other protein assays have been used to assess TJP abundance and expression levels. Although less commonly used, co-immunoprecipitation has been utilized to evaluate the abundance of specific protein interaction complexes [90]. PCR has been employed to measure changes in the TJP gene expression. Notably, differences between protein and mRNA expression levels can provide insight into regulatory mechanisms. In some cases, a reduction in mRNA, but not membrane protein, expression may indicate that repair mechanisms have been engaged [91].

Sample preparation must also be considered when evaluating TJPs. In the CNS, TJPs are expressed at much higher levels in the microvessel ECs, but their expression in other cell types should not be ignored. In addition to CLDN-11 in the myelin sheath, OCLN expression in primary neuron and astrocyte cultures has been noted [92,93]. Further, CLDN-1 and -5, as well as ZO-1 and -2, have been reported in peripheral blood leukocytes from healthy human patients [94]. Thus, microvessel isolation should be completed to avoid inclusion of TJPs linked to other cell types.

Studies examining changes in BBB permeability over time have revealed effects that are lasting and may vary in a biphasic manner. For example, focal ischemia in rats produces a biphasic increase in BBB permeability at 8 and 120 h, which is accompanied by a reduction in OCLN and CLDN-5 protein expression [95]. Likewise, inflammatory pain can have a biphasic effect on BBB permeability linked to variation in the expression of TJPs [96]. Increased BBB permeability was reported at 1–6 h and again at 48 h after pain, which coincided with increased ZO-1 expression and decreased OCLN expression [96]. Other neural insults have been shown to have a lasting effect. For example, cortical compression in rats increases extravasation of Evan’s blue 3 days later [97]. While no acute changes in TJP expression were reported, there was a significant increase in the expression of OCLN and CLDN-5 for 1–2 weeks following cortical compression [97]. The findings show that neural injury and inflammation can impact TJP expression beyond the immediate acute and subacute phases.

## 4. BBB Development and Function

Descriptions of the BBB often characterize it as a static structure that serves to protect the CNS from peripheral factors. However, the barrier is a dynamic structure that changes over time and responds to cues originating from both the periphery and the CNS. It allows the entrance of nutrients, the efflux of waste, and facilitates communication with the rest of the body through systemic factors. Here, ECs lack pinocytic activity and fenestrations, preventing diffusion and passive exchange of molecules between the tissue and bloodstream. As a result, active transport mechanisms are needed to deliver nutrients and substrates into the CNS.

### 4.1. The BBB Develops Early but Is Not Immediately Functional

During neural development, angiogenesis and neurogenesis occur in close synchrony, but the formation of a functional barrier is delayed [55,56]. A tip cell, which is a specialized EC, forms capillaries through the developing parenchyma under the direction of guidance cues such as VEGF, Notch, Ephrin, PDGFRB, and Wnt/β-catenin. In addition, neurons release factors that promote angiogenesis, while ECs release factors that promote further synaptogenesis, neural proliferation, and development [55].

**Figure 4:**
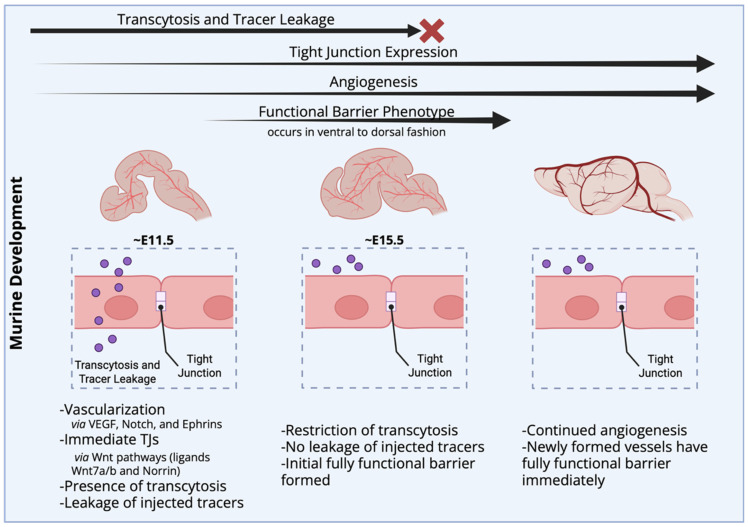
Functional and developmental timeline of the BBB. Vascularization occurs early in development due to the expression of angiogenic factors. At this time, TJs between ECs are present, but injected tracers still pass the barrier, and transcytosis occurs. There is a later restriction of transcytosis and absence of tracer leakage, which is considered the full functional barrier phenotype. As vascularization continues through development, new vessels form with fully functional barriers. Created with Biorender.com.

To elucidate the factors that contribute to the development of the EC vasculature, researchers have transplanted neural tissue that had not yet been vascularized into the coelom of a host [52,55,56,57]. In this paradigm, the host organism provides the vascularization into the neural tissue. Blood vessels developed in the neural graft and displayed BBB-like characteristics (no leakage of trypan blue, indicating the presence of TJs). Importantly, non-neuronal tissue transplanted into host ventricles lacked BBB characteristics once vascularized. The results suggest that the neural environment prompts ECs to differentiate and form the blood–brain barrier [57]. As development continues, the expression of factors specific to the CNS results in the formation and later stabilization of TJs. After this, there is a restriction of transcytosis, creating a highly impermeable barrier [55,56]. An overview of the functional development of the BBB is provided in Figure 4.

### 4.2. Alterations in BBB Function with Aging

Once formed, the BBB changes in a dynamic manner, particularly with aging. This process is of interest because age-related alterations in the BBB may contribute to neurodegeneration [34,35,54,98]. A key issue is whether increased permeability fosters the development of neurodegenerative disorders such as Alzheimer’s disease and amyotrophic lateral sclerosis—or does neurodegeneration lead to the breakdown of the BBB? Age-related changes in the BBB are also of interest because older individuals suffer a higher incidence of neurotrauma [99,100].

At a mechanistic level, studies in mice have shown that older animals have increased ligand-specific mediated transport across the BBB and less specific caveolae-dependent transport [61]. In humans, DCE-MRI has revealed increased BBB permeability in aged individuals [101]. Interestingly, these increases in BBB permeability were most prominent in brain regions associated with higher-order cognition, suggesting that age-related barrier disruption could contribute to cognitive decline [101]. In addition to functional consequences, age-related changes may make the aged brain more susceptible to certain compounds. For example, administration of tissue-type plasminogen activator (tPA), a common treatment for ischemic stroke, increases barrier permeability, and this is amplified in older mice [62]. Due to the interaction of age and barrier function, future research should consider age as a factor when exploring barrier disruption in pathological conditions.

### 4.3. Waste Clearance

Because the barrier restricts the efflux of waste as well as the influx of nutrients, processes are needed to clear cellular debris. Furthermore, clearance is implicated in many neurological diseases, as the buildup of cellular waste can be toxic [98]. Misfolded proteins are one of the most common cellular waste by-products. Aggregation of misfolded proteins has been implicated in several diseases, including Alzheimer’s disease, Parkinson’s disease, and TBI [98]. There is also a need to transport other waste products, such as lactic acid, which is produced by astrocytes and used by other cell types (e.g., neurons). Likewise, released neurotransmitters must be cleared through diffusion, enzymatic degradation, neuronal reuptake, or uptake by glial cells. When these mechanisms go awry, the buildup of excitatory neurotransmitters (e.g., glutamate) can lead to pathology [49,50,98]. Indeed, glutamate excitotoxicity can promote cellular degeneration, fueling motor dysfunction, paralysis, and epilepsy.

Parenchymal waste clearance is thought to occur through two pathways, the CSF pathway and the vascular pathway [98]. The glymphatic system and intramural periarterial drainage (IPAD) are two distinct mechanisms that have been proposed to explain how waste can exit the parenchyma through the CSF. The glymphatic pathway involves arterial pulses and aquaporin-4 activity, which produce a flow of CSF into the parenchyma. Here, CSF mixes with interstitial fluid and waste before exiting through the perivenous space. IPAD involves a unidirectional flow in which interstitial fluid passively flows along the basement membrane, relying on efflux of waste from the parenchyma. Although debated, it is possible that both the glymphatic system and IPAD contribute to waste clearance through the CSF pathway.

The vascular pathway involves the efflux of waste directly into the bloodstream; however, this is highly selective due to the presence of TJs. Waste can be transported to the blood passively through paracellular transport (limited by the BBB) and transcellular transport (movement of small molecules such as water and ethanol), or actively through transporters or transcytosis [98].

Active transport mechanisms include receptor-mediated, carrier-mediated, and adsorptive-mediated transport. An example of receptor-mediated transport is provided by insulin, which is transported by insulin receptors on ECs. Carrier-mediated transport is a significant contributor to the delivery of nutrients to the CNS. Examples include glucose transporters that provide fuel for the CNS and amino acid transporters used to deliver the building blocks for proteins [98,99,100].

### 4.4. Endocrine Function

Another dynamic feature of the BBB is its ability to act as both an endocrine target tissue and a secretory tissue, while also allowing the enclosed tissue to do the same [26,27,28]. In vitro models have demonstrated the potential for brain ECs to display polarized constitutive secretion of cytokines under unstimulated conditions [28,29]. Challenges with lipopolysaccharide (LPS) applied to either the luminal or abluminal side of the monolayer led to increased cytokine secretion. Notably, the secretion of many cytokines was increased on both sides of the monolayers [28,29]. While some experiments have utilized EC-only cultures, others have employed tricultures to explore the involvement of pericytes and astrocytes, which exhibit enhanced secretion of several proinflammatory cytokines [28]. The findings show that the BBB can receive inflammatory signals from both internal and external environments and produce a response that can be transduced to either environment.

The endocrine-like function of the BBB can lead to alterations in BBB permeability in response to factors in the peripheral circulation. Mood disorders, such as post-traumatic stress and major depressive disorder, increase levels of circulating proinflammatory cytokines, and these increases have been correlated with increased barrier permeability (see review [101]). Furthermore, there is evidence linking TJP expression, mood disorders, and proinflammatory cytokine infiltration. Loss of CLDN-5 has been observed in post-mortem samples from depressed patients as well as stress-susceptible mice [102]. This downregulation of CLDN-5 in mice was associated with increased infiltration of interleukin 6 (IL-6) into the brain. The links between stress, mood disorders, peripheral inflammation, and barrier disruption have been extensively studied (see below and [103,104]).

## 5. Factors External to the BBB That Affect Permeability

**Figure 5:**
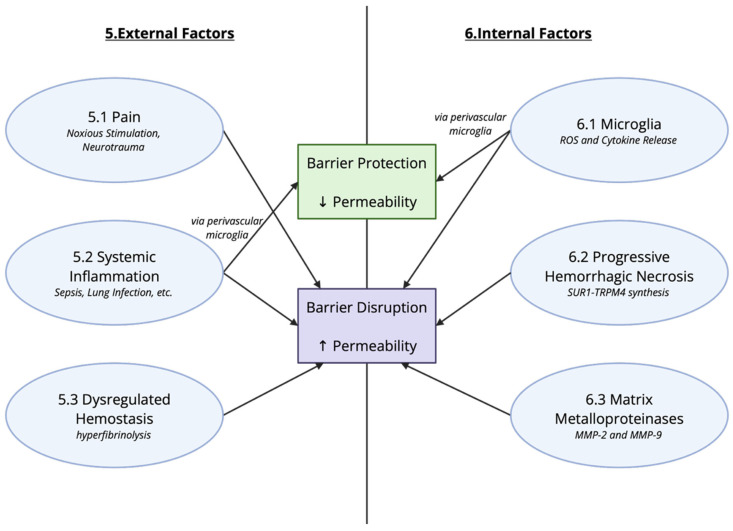
Overview of internal and external factors affecting barrier permeability. The current review will focus on external and internal factors that disrupt the BBB, causing an increase in permeability. Arrows indicate processes that lead to changes in barrier integrity. In some cases, inflammation and microglial activation may have a protective effect that reduces permeability. Created with Biorender.com.

Given that the BBB/BSCB separate two compartments of the body, central versus peripheral, there is a unique opportunity to classify events leading to disruption of the barrier as either internal or external, a concept used by da Fonseca to describe changes in barrier function in regard to epilepsy [52]. This section will focus on insults to barrier function in which the initial precipitating event occurs outside of the CNS. It is important to acknowledge that the majority, if not all, extrinsic factors that a vertebrate organism encounters will produce some change in the CNS. Pain, systemic inflammation, and infection are all exogenous factors that can negatively impact barrier function. Figure 5 summarizes the key factors contributing to changes in barrier functions.

### 5.1. Pain Can Produce a Breakdown of the CNS Barrier

It is known that pain can increase BBB permeability, and some have speculated that this may be linked to changes in TJP expression [73,91,96,105,106,107]. The International Association for the Study of Pain defines pain as a sensory and emotional experience [108], whereas noxious stimulation refers to input to the nervous system that is tied to actual or potential tissue damage [109]. It is important to note that pain often arises due to noxious stimulation, but pain can occur without noxious stimulation and vice versa.

A variety of preclinical techniques have been developed to study factors that influence the development of enhanced pain, many of which have also been implicated in BBB disruption. Inflammatory pain can be modeled using systemic or local injection of irritants like carrageenan, capsaicin, and complete Freund’s adjuvant (CFA) [73,96]. Stimulation of pain fibers with electrical stimulation can also be used; however, this is often not as selective as chemical agents because multiple fiber types (e.g., C and A-δ fibers) involved in pain (nociceptive) sensory signaling may be engaged. Peripheral nerve injury is another common pain paradigm, used to model neuropathic pain through chronic constriction and spared nerve injuries [91,107]. Central pain models involving neural injury are also associated with chronic pain [4,110,111]. While many have shown these pain models to induce disruption of CNS barriers, the particular parameters and mechanisms responsible for the disruption are not well-understood.

Carrageenan paw injections have been shown to produce increased extravasation of [14C] sucrose into the parenchyma with peaks at 3 h and 48 h after injection [96]. OCLN expression decreased along a similar time course. The authors showed that there are two corresponding phases in the expression of OCLN bands; the β band demonstrates a decrease in expression during the initial phase (0–6 h), and the α band shows a decrease in expression during the second phase (12–72 h), which is also accompanied by an increase in the β band [96]. This differential expression of the α and β bands of OCLN over time may indicate that two processes drive the increase in BBB permeability. In addition, ZO-1 expression was significantly increased 1 h after injection and slowly dropped until returning to control levels [96].

Others have explored barrier function after the induction of inflammatory pain using CFA paw injections. Animals displayed reduced withdrawal latency to a heat source as well as increased edema at 24, 48, and 72 h [73]. Increased extravasation into the parenchyma was observed when using [14C] sucrose but not Evan’s blue. Considering the greater size of Evan’s blue molecules relative to [14C] sucrose, this result suggests that there is a mild barrier disruption in response to inflammatory pain. At the latest time point, brain microvessels were isolated. Animals treated with CFA displayed increased expression of CLDN-3 and -5, decreased OCLN, and no changes observed in actin or ZO-1 relative to vehicle (saline)-treated controls [73]. Immunofluorescent imaging revealed that the expression of OCLN was strand-like in saline-treated animals as opposed to punctate and bead-like in the CFA-treated animals. Overall OCLN expression was also lower in CFA-treated animals. These studies suggest that inflammatory pain leads to barrier disruption by altering expression of TJPs.

Exposure of ECs to inflammatory substances could directly damage the cells and produce barrier breakdown. To evaluate direct effects of inflammatory agents, carrageenan, CFA, or formalin were applied in vitro to bovine brain microvessel EC monolayers [112]. With the exception of high-concentration formalin, none of the inflammatory agents affected EC viability or permeability to [14C] sucrose. Additionally, after isolating brain microvessels, no changes were observed in protein expression of OCLN, ZO-1, actin, or CLDN-1 in any conditions [112]. This suggests that nociceptive activity, not the presence of inflammatory substances, may mediate CNS barrier disruption.

While it is clear that inflammatory pain increases barrier permeability, it remains unclear whether BBB breakdown is related to nociceptive neural activity or the associated expression of inflammatory factors. To explore these issues, researchers combined a carrageenan paw injection with a peripheral nerve block that would diminish the consequent neural activity [105]. They found that carrageenan injection decreased paw withdrawal latency to a heat source, increased edema formation in the injected paw, and increased extravasation of [14C] sucrose. Bupivacaine (a local anesthetic) applied to the ipsilateral saphenous, tibial, and common peroneal nerves attenuated hyperalgesia and the increase in BBB permeability but not edema formation. The results suggest that the alteration in pain and BBB permeability is driven by the sensory signal, whereas edema is tied to the inflammatory response. Carrageenan treatment also increased protein expression of ZO-1, CLDN-5, and OCLN 1 h after injections, and these effects were attenuated by the nerve block [105]. Given that TJPs are traditionally implicated in the maintenance of an impermeable barrier, their upregulated expression is surprising. Increased expression could reflect the engagement of a compensatory process that fails to maintain BBB integrity.

Other work suggests that neuropathic pain can increase BBB permeability as well as TJP expression and colocalization. Peripheral nerve injury from either chronic constriction injury or spared nerve injury induced an increase in horseradish peroxidase extravasation in the spinal cord of animals relative to sham-operated controls [107]. The duration and extent of HRP extravasation varied slightly by neuropathy model but generally increased by 24 h and remained elevated as late as 3 days. This group, like others, found that blocking the afferent nociceptive signals with lidocaine (a local anesthetic) prevents the breakdown of the BSCB [107].

Researchers have also explored the effect of treatments that directly drive nociceptive fibers [107]. Electrical stimulation increased BSCB permeability to Evan’s blue when applied to sensory afferents at a higher intensity that engaged both unmyelinated C-fibers and A-δ fibers, but not at lower intensities that only engaged A-δ fibers. Afferent fibers that express the transient receptor potential cation channel subfamily V member 1 (TRPV-1) receptor were implicated by showing that the TRPV-1 agonist capsaicin applied to the sciatic nerve caused an increase in BSCB permeability 24 h later. This breakdown of the BSCB was concentrated in the spinal cord but extended throughout the CNS with less extravasation in the BBB further from the stimulation site. While the relative impact of capsaicin on BSCB versus the BBB differed, this could reflect differences in proximity to the stimulation site rather than variation in barrier function. In addition to the plasma extravasation into the CNS, capsaicin treatment induced peripheral plasma extravasation that was visually apparent 45 min later [107].

Sauer et al. (2017) [91] provide a broad overview of BSCB and TJP changes in mice following spared nerve injury, as well as in rats following chronic constrictive injury [69]. Both injury types produced enhanced pain. Membrane protein expression was largely unchanged, except for OCLN, which was decreased at 7 and 14 days following injury in rats. Conversely, several TJPs were shown to have downregulated mRNA expression, including *claudin-1*, *-19*, *tricellulin*, *occludin*, and *ZO-1* in rats at 14 days following injury, as well as *claudin-5*, *-19*, *tricellulin*, *occludin*, and *ZO-1* mRNA in mice at 7 days post-injury. The authors suggest that this discrepancy in the membrane protein and mRNA expression may reflect slowed protein degradation and continued translocation of TJPs to the membrane following injury [91].

Sauer et al. (2017) [91] also evaluated pericytes using a PDGFRB marker and found fewer PDGFR-β+ cells 7 and 14 days following a chronic constriction injury. In addition, β-catenin, which can promote TJP expression and adherens junction stabilization, was decreased 7 days after injury. The authors point out that PDGFRB and transmembrane proteoglycan NG2 are both markers for pericytes. NG2 is also a substrate for MMP-9, which is known to trigger BBB/BSCB breakdown, increase ROS production, and engage other processes detrimental to neuronal survival [91]. The findings suggest that neuropathic pain can undermine barrier function by disrupting pericytes in the NVU and altering expression of TJ proteins and mRNA.

While both inflammatory pain and peripheral nerve injury have been shown to disrupt the BBB, others have shown that nociceptive stimulation drives barrier disruption following SCI [3,4,111]. Recently, a similar effect has been reported after TBI, providing an example of how treatments can have similar effects on the BSCB and BBB [113]. Noxious tail shock or capsaicin paw injections administered after SCI increased hemorrhage at the site of SCI. In addition to promoting barrier disruption, nociceptive stimulation amplified injury-induced locomotor deficits. Infusion of lidocaine rostral to the injury attenuated both the increased hemorrhage and locomotor deficits. General anesthesia was shown to have a similar protective effect. These findings again implicate neuronal signaling. Furthermore, the observation that blocking communication with the brain, or general anesthesia, prevents pain-induced hemorrhage suggests that brain-dependent processes may foster the breakdown of the BSCB [111].

In summary, nociceptive activity has been shown to promote the breakdown of the BBB. This effect has been linked to activity in unmyelinated C fibers and afferent fibers that express the TRPV1 receptor. While there is evidence that activation of myelinated A-δ fibers does not foster hemorrhage, further work is needed using procedures that enable the selective engagement of this fiber type. Functional alterations in barrier permeability are accompanied by changes in TJP expression, localization, and organization. The adverse effect of noxious stimulation can be attenuated by treatments that disrupt communication with rostral neural systems, implicating brain-dependent processes. Clearly, nociceptive signaling can adversely affect barrier function, and this presents a clinically relevant therapeutic target.

### 5.2. Systemic Inflammation Contributes to Barrier Disruption

While the primary purpose of the BBB is to exclude circulating factors that are neurotoxic from the CNS, systemic inflammation from infection, autoimmune disease, or trauma can still have effects on the CNS and the barrier itself [11,114]. The expression of adhesion molecules within the lumen formed in brain microvessels provides a mechanism by which systemic inflammatory signals can be transduced into the CNS to affect the barrier and the nervous tissue [115]. Preclinical models of inflammation come in a wide variety, including systemic injections of inflammatory agents like carrageenan or LPS, inoculation with pathogens, or cecal ligation and puncture [116,117]. Clinical studies have shown that traumatic injuries are associated with increased incidence of infection, complications, and mortality [114].

Researchers have developed models to replicate common comorbid complications seen in patients. Exposure to various pathogens, such as streptococcus pneumoniae or staphylococcus aureus, can be used to model hospital-acquired infections frequently linked to ventilator dependence after acute neurotrauma [114,118,119]. The use of puncture and/or ligation of bile ducts in the abdomen is used to model sepsis, another common complication of severe trauma [118].

A study examining systemic inflammation induced by daily LPS injections (i.p.) revealed distinct roles for activated microglia [63]. In the acute period, microglia are recruited to the vessel where they physically invade the NVU. This was associated with decreased BBB permeability. Conversely, after several days of systemic LPS, microglia phagocytosed astrocytic end feet, which increased BBB permeability [63]. These data suggest that systemic inflammation alters the BBB via CNS responses, and the duration of inflammation can influence the nature of this effect.

Others have utilized pseudomonas aeruginosa lung infection to model systemic inflammation, which resulted in increased BBB permeability as well as permeability of lung epithelia. These changes were observed up to 7 days following infection. The shift in permeability was accompanied by decreased *VE-cadherin* and *claudin-5* mRNA, as well as decreased VE-cadherin, OCLN, and CLDN-5 protein expression. Immunofluorescence revealed disorganization in the expression patterns of ZO-1, CLDN-5, and VE-cadherin. Despite functional disruption and the accompanying molecular changes, no pseudomonas aeruginosa bacteria were found in the brain parenchyma. Nonetheless, lung infection increased the expression of IL-1β, IL-6, TNF-α, CXCL1, CXCL2, and ICAM-1 in the cortex and hippocampus [120]. This supports the conclusion that, while barrier disruption is caused by infiltration of peripheral components, the brain can detect and respond to inflammatory signals without entry of the insulting vector itself.

Studies examining the effect of sepsis-associated encephalopathy provide further evidence that systemic inflammation can lead to BBB disruption [121]. A mouse model of cecal ligation and puncture produced sepsis-induced cognitive deficits, increased BBB permeability, microglia and astrocyte activation, elevated brain and serum proinflammatory cytokine (TNF-α, IL-6, and IL-1β) expression, and increased high-mobility group box 1 (HMGB1) and the receptor for advanced glycation endproducts (RAGE) protein expression. The authors hypothesized that agonizing the α2 adrenoceptor within the locus coeruleus would attenuate these effects. Treatment with dexmedetomidine, a highly selective α2 adrenoceptor agonist commonly used for sedation in intensive care units, attenuated neuroinflammation, barrier disruption, and cognitive deficits. Further, BRL-4440, a selective α2A antagonist, blocked the protective effects of dexmedetomidine. The authors proposed that systemic inflammation triggers the release of proinflammatory cytokines and HMGB1, which activate ECs via RAGE and increase barrier permeability. Treatment with an α2A adrenoceptor agonist mitigates these effects, suggesting a role for systemic inflammation in both barrier disruption and neuroinflammation [121].

Other work has implicated damage-associated molecular pattern HMGB-1 and pattern recognition receptor RAGE in barrier disruption [122]. Surgical trauma from a splenectomy procedure in aged mice increased IgG extravasation and disrupted TJ structure in the hippocampus relative to naive controls. TJs increase swelling and perivascular space, as well as swollen and sometimes detached astrocytic end feet. These changes were associated with increased proinflammatory cytokines (TNF-α and IL-1β) as well as HMGB1 and RAGE protein and mRNA. On a parallel time course, splenectomy-treated animals exhibited deficits in spatial memory. In non-surgery anesthesia-treated controls compared to naive animals, these changes were still observed, but on a milder scale with a shorter time course [122]. While not a direct manipulation of systemic inflammation, surgery and possibly anesthesia treatment produce an inflammatory response. Furthermore, HMGB1 and RAGE have been implicated in neural injury as well [123]. Animals subjected to a TBI and treated with a neutralizing monoclonal antibody for HMGB1 showed significant improvements in lesion size, neuronal death, and barrier disruption within the first 24 h. The treatment also attenuated the expression of MMP2, MMP9, and TNF-α, all of which have been associated with barrier disruption [123]. The results suggest that HMGB1 and RAGE play a pivotal role in inflammation-induced BBB disruption after neural injury.

A clinical study examining patients with COVID-19, particularly long COVID-19, found evidence of barrier dysfunction associated with cognitive deficits [124]. While nearly all patients with COVID-19 displayed increased proinflammatory cytokines and coagulopathy factors in blood serum, patients with brain fog had significantly increased S100B, GM-CSF, and IL-6 levels. These patients also exhibited greater BBB disruption as measured by DCE-MRI. This barrier alteration had a moderately strong positive correlation with plasma levels of TGF-β [124]. While it is not known if COVID-19-induced inflammation and BBB disruption produced the cognitive deficits, their co-occurrence warrants additional investigation.

Taken together, there is considerable evidence that systemic inflammation can lead to BBB disruption. There is a high incidence of concurrent infections in neurotrauma survivors, both in the acute and chronic phases of injury [114]. Some have argued that this is due to a rapid peak in the inflammatory response, which is ultimately exhausted, leaving the system compromised and predisposed to infection. In a preclinical model, TBI and pneumonia alone caused pulmonary edema and increased proinflammatory cytokine expression. Notably, when blood samples from TBI or sham-treated animals were challenged ex vivo, samples from subjects with a TBI had reduced TNF-α production [119].

Other groups have shown similar immunosuppressive effects of neural injury and subsequent changes in motor function [118]. Animals subjected to streptococcus pneumoniae (Sp) following TBI had increased mortality relative to all control groups and increased motor deficits compared to TBI-only controls. Interestingly, unlike the behavioral effects, the extent of inflammatory response in the lungs varied with the time between neural injury and Sp exposure. Specifically, when Sp exposure occurred in the acute phase, lung tissue from TBI animals had comparable levels of proinflammatory cytokines and a reduction in TNF-α compared to sham animals. When Sp exposure occurred in the chronic phase (60 days post-injury), TBI animals displayed increased IL-1β and TNF-α in lung tissue compared to sham-treated controls. The findings suggest that neural injury can disrupt the systemic immune response, and that the nature of this disruption varies with the time between injury and pathogen exposure. Increased neuroinflammation was also observed in TBI animals with Sp exposure compared to TBI-only controls, regardless of when exposure occurred [118]. These data suggest that neural injury causes widespread immunosuppression, which can augment neuroinflammation during both the acute and chronic phases of recovery.

Systemic inflammation can be triggered by several factors, including infection, sepsis, surgical trauma, and neurotrauma, all of which were shown to promote disruption of the CNS barriers. It seems inflammatory cytokines and damage-associated molecular patterns promote the degradation of TJPs, resulting in increased barrier permeability. In addition to altering barrier permeability, systemic inflammation results in increased cognitive deficits as well as injury-specific functional deficits when combined with neurotrauma injuries. The feedback loop between systemic and neural inflammatory responses results in unchecked inflammation that can increase mortality. Reducing neuroinflammation may be protective against systemic infections, while managing systemic infections may be protective against neural injuries.

### 5.3. Regulation of Coagulation and Fibrinolysis Is Important to Barrier Function

**Figure 6:**
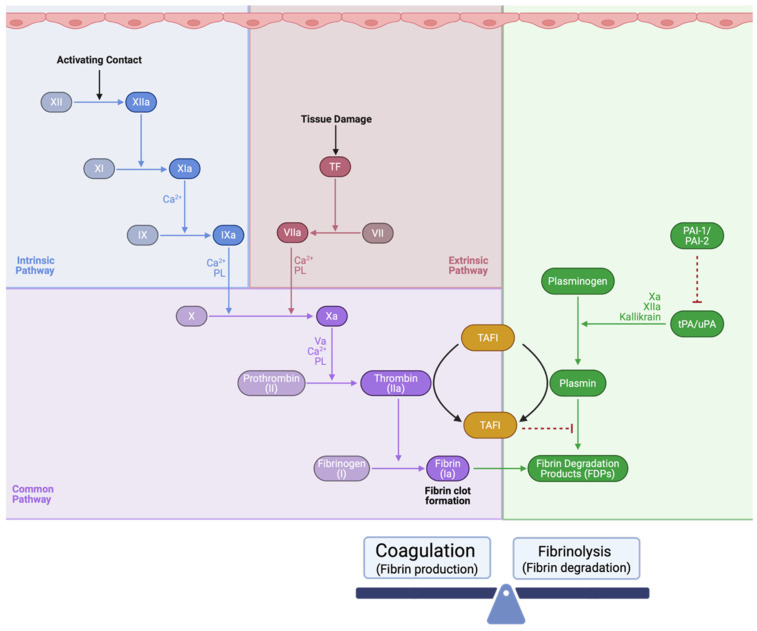
Coagulation and fibrinolytic cascades. The signaling cascades mediating coagulation and fibrinolysis are represented here. The intrinsic and extrinsic pathways converge to the common pathway, ultimately leading to fibrin production. The fibrinolysis cascade is responsible for fibrin degradation. Many of these molecules interact with the BBB. Note: Adapted from “Data supporting the structural and functional characterization of Thrombin-Activatable Fibrinolysis Inhibitor in breast cancer” by M.S. Fawzy and E.A. Toriah, 2015 [127], *Data in Brief*, page 983. Created with Biorender.com.

Two mechanisms have been implicated in maintaining hemostasis of the blood: coagulopathy and fibrinolysis [125]. Coagulopathy refers to clot formation in response to damage to the endothelium and/or tissue injury. Clot formation is needed to reduce bleeding and acute damage, but eventually the clot must be disrupted to prevent thrombosis. Fibrinolysis is the physiological mechanism responsible for the degradation of the formed clot [125]. Figure 6 illustrates the coagulation and fibrinolytic cascades. Disruption of the balance between these processes can result in exacerbation of tissue loss and, in the setting of neural injury, increase morbidity and mortality. Furthermore, it is important to note the multifaceted nature of these processes and how outcomes can interact. On the one hand, problems may arise from increased clot formation due to hypercoagulopathy and/or hypofibrinolysis [125,126]. On the other hand, hypocoagulopathy and/or hyperfibrinolysis can fuel hemorrhage.

The coagulation cascade is the process by which fibrin clots are formed [126,127]. Traditionally, this process has been classified into intrinsic (contact-activated) and extrinsic (tissue injury-activated) pathways, both of which ultimately converge to form the common pathway. The fibrinolytic cascades degrade fibrin clots via plasminogen activators [125,126,128]. Thrombin and plasmin, components of this cascade, activate thrombin-activatable fibrinolysis inhibitor (TAFI). Activated by both fibrinolysis and coagulation cascades, TAFI stabilizes clots by limiting plasmin production [125,126,128]. Beyond hemostasis, these signal cascades also influence neuroinflammation and barrier functions. For example, tPA is known to disrupt barrier function by promoting phosphorylation of CLDN-5 and altering OCLN expression [62]. This effect was associated with increased incidence of hemorrhagic transformation, particularly in aged animals. Fibrinogen has been shown to induce changes in heart microvascular cells and has been implicated in increased permeability to albumin, reduced TEER, and reduced protein and mRNA expression of OCLN, ZO-1, and ZO-2 [129]. This effect is thought to be due to fibrinogen binding to ICAM-1 receptors on ECs, engaging MEK/ERK pathways and driving downstream effects, such as f-actin formation and disassembly of TJPs [129]. Both plasminogen and plasmin have been shown to promote proBDNF conversion to its mature form and fibrinogen degradation in the presence of astrocytes [48]. Thrombin infused unilaterally into the basal ganglia of rats at doses comparable to those resulting from intracerebral hemorrhage increased BBB permeability in the ipsilateral cortex 24 h later [130].

Research by Hijazi et al. (2015) [131] implicated tPA and fibrinolysis in intracerebral hemorrhage following a closed-head TBI in mice. A catalytically inactive tPA variant was used to inhibit endogenous WT tPA binding to fibrin, leading to reduced clot lysis induced by WT tPA or uPA in a dose-dependent manner. TBI led to increased hemoglobin in the brain of WT animals, and this effect was attenuated by post-injury IV administration of the variant tPA. KO of tPA and uPA similarly reduced hemoglobin, fibrinolytic activity, and neurological severity scores 24 h post-injury. The tPA variant had no effect on KO animals unless WT tPA or uPA was reintroduced. KO of plasminogen activator inhibitor-1 (PAI-1), an endogenous inhibitor to tPA and uPA, worsened hemorrhage, and this was again attenuated by the tPA variant [132]. Notably, tPA is fibrin-dependent, whereas uPA is not [133]. tPA KO mice showed post-TBI increased D-dimer and decreased platelet counts, which are markers of fibrinolysis, while uPA KO mice did not. These findings support a role for tPA and uPA in TBI-induced hemorrhage linked to hyperfibrinolysis.

Researchers have explored whether barrier disruption may be related to noncatalytic effects of tPA [132]. After observing the protective effects of inhibiting tPA with the PAI-1, fragment peptides from PAI-1 were developed to assess noncatalytic effects. One modified peptide interacted with the PAI-1 binding site on tPA and had no effect on fibrinolysis or clot degradation. This peptide reduced tPA-induced BBB disruption and mortality, and improved neurological severity scores following both ischemic and thromboembolic stroke. Developed to increase the therapeutic window of administration for exogenous tPA therapy in the treatment of stroke, this peptide could protect the BBB from endogenous tPA released after injury [132]. Although further investigation is needed, the peptide may limit progressive barrier disruption without transitioning the system to a hypercoagulable state.

Given the delicate balance between clot formation and degradation, coagulopathy and fibrinolysis cascades provide ample opportunity to modulate barrier function in both physiological and pathological settings. Dysregulation of coagulopathy and fibrinolysis is believed to occur after neural injury, but can be difficult to identify and treat acutely due to heterogeneous presentation and laboratory diagnostics, as well as the interactions of concurrent injuries and treatments [126]. There are a number of assays, such as platelet count, prothrombin time (PT), activated partial thromboplastin time (aPTT), international normalized ratio (INR), and viscoelastic hemostatic assays (VHA), that are commonly used in the clinical assessment of hemostasis. Unfortunately, these measures have significant limitations, including time from collection to actionable results, disagreement regarding defining thresholds, and the potential to yield values that appear ‘abnormal’ even under normal clotting conditions.

Although not observed in all patients, both fibrinolysis and coagulopathy can promote hemorrhage and death. It is not clear what causes the presentation of a hypercoagulable versus a hyperfibrinolytic profile following trauma. Hyperfibrinolysis during the early acute phase leads to uncontrolled hemorrhage and higher mortality. Conversely, unchecked coagulation, often in more chronic phases, results in thrombosis, which also increases mortality [126]. While there are instances in which treatment can minimize these effects, further research is needed to identify the underlying factors that contribute to the various phenotypes of blood hemostasis to derive standard treatments for differential presentations.

## 6. Alterations in BBB Permeability Due to Local Factors

The preceding section focused on external/peripheral factors that can impact BBB/BSCB integrity and permeability. We now shift our attention to cellular events that impact barrier function from the other side: intrinsic factors that can trigger a breakdown of the BBB. As we will see, many of these are again linked to injury and inflammation.

### 6.1. Microglia Modulate Barrier Function via Proinflammatory Factors and Phagocytosis

Microglia have been shown to affect the function and permeability of the BBB. Their activation results in processes such as ROS production, which can affect BBB integrity through changes in TJP organization [134]. Utilizing rat and human EC lines, Schreibelt et al. (2007) demonstrated that ROS treatment resulted in decreased TEER, increased permeability to a 150 kDa tracer, and increased stress bundle formation (F-actin formation) [134]. Live-cell confocal microscopy revealed that ROS treatment disrupted the expression of CLDN-5 and OCLN at the plasma membrane. The authors linked this to the phosphorylation of Rho, Rho A, PI3K, and PKB pathways using the respective inhibitors, toxin B, C3 transferase, wortmannin, and triciribine. In each case, there was reduced monocyte transendothelial migration following ROS treatment. It is clear that ROS negatively affected the functional permeability of the microvascular barrier, possibly through changes in EC expression of TJs mediated by the Rho/PI3k/PKB pathway [134].

Sumi et al. (2010) showed that reducing the ROS production and release from microglia with diphenyleneiodonium chloride, a NADPH oxidase (NOX2) inhibitor, had a reparative effect [135]. Co-cultures of microglia and rat brain microvascular ECs resulted in decreased TEER and increased permeability when challenged with LPS, while EC-only cultures demonstrated none of these changes. Additionally, immunofluorescent staining revealed disrupted TJP organization, particularly of ZO-1 and CLDN-5. Interestingly, there was no change in protein expression levels associated with this effect. The authors suggested ROS production from microglia led to TJP disorganization and increased barrier permeability. The lack of response to LPS treatment in EC cultures suggests that the inflammatory response from microglia mediates the breakdown of the barrier rather than being a direct effect of LPS on ECs themselves [135].

Others have provided in vivo evidence that blocking ROS production from microglia has a protective effect in neurotrauma [136]. Advanced oxidation protein products (AOPPs), considered a marker of oxidative stress, are thought to be both products and activators of ROS. A unilateral hemisection SCI was shown to induce oxidative stress within 3 days, assessed by CSF and plasma AOPPs as well as dihydroethidium in parenchyma. Daily systemic treatment with apocynin, a NOX2 inhibitor, significantly attenuated these effects and microglial activation, and improved long-term recovery of motor function at 28 days [136]. Others have implicated NOX2 with KO models or treatment with NOX2ds-tat following SCI, which resulted in a long-term improvement in hindlimb locomotor function, reduced behavioral signs of chronic pain, increased white matter sparing, and increased neuronal counts following a thoracic contusion SCI [137]. Expression of *TNF-α*, *IL-1β*, *iNOS*, and *IL-6* genes was upregulated following SCI, and this too was attenuated in NOX2 KO animals. Furthermore, these effects were associated with reduced ROS production from activated microglia [137]. There is ample evidence for increased ROS production as a result of microglia activation and the negative effects of this process in neural injury. While these studies did not directly assess the effects of blocking microglial ROS production on barrier permeability, the findings suggest that ROS production leads to increased neuroinflammation and worsens functional outcomes, both of which may be tied to barrier disruption. Targeting microglia to reduce ROS production could help preserve barrier integrity by limiting the spread and duration of neuroinflammation following neurological injury.

Other groups have implicated microglial-associated production and release of cytokines. Of particular interest, fibrinogen and serum albumin have been shown to increase the secretion of cytokines from microglia [138]. Exposure of microglia cultures to these blood products increased the expression of proteins known to disrupt the BBB (e.g., IL-1β, IL-6, IL-10, TNF-α, and MCP-1). Increased expression of MIP-1α was also observed and has been associated with increased immune cell migration into the CNS. Fibrinogen and albumin exposure increased microglia migration, providing a mechanism to perpetuate barrier breakdown from even minor insults [138]. This produces a cycle in which disruption leads to blood product exposure, which further exacerbates neuroinflammation and subsequent barrier disruption.

Using a model of the rat BBB, LPS-stimulated microglia were shown to be the primary source of secreted TNF-α, IL-1β, MIP-1α, and IL-1α [139]. Furthermore, microglia interacted with other cells within the NVU, particularly astrocytes, to promote the release of additional chemokines and cytokines. In addition to TNF-α and IL-1β secreted by microglia, astrocytes co-cultured with LPS-stimulated microglia secreted MCP-1 and IL-6, both of which are known to promote barrier disruption. These changes in chemokine and cytokine secretion were associated with reduced TEER, increased permeability, and reduced protein expression of ZO-1 and OCLN [139].

Apoptosis signal-regulated kinase 1 (ASK1) and c-Jun N-terminal kinase (JNK) have been implicated in microglial and EC activation, both of which result in BBB/BSCB alterations [140]. Meng et al. (2023) explored the role of ASK1 using a transgenic mouse line with a point mutation (ASK1-K716R) that yields an inactive form of ASK1 [140]. This mutation-attenuated TBI induced increases in p-ASK1 and p-JNK in brain parenchyma 3 days post-injury. While there was no change in the overall number of activated microglia, the cells exhibited a shift to a more anti-inflammatory and less proinflammatory state. The mutation also resulted in an overall reduction in the number of apoptotic ECs and reduced OCLN, VE-cadherin, and MMP-9 protein expression. On a functional level, the BBB appears to be protected following TBI in ASK1-K716R mutated mice, with reduced leakage of exogenous and endogenous molecules in the mutant animals following TBI compared to WT counterparts. At 35 days post-injury, there was reduced white matter loss in ASK1-K716R mutants compared to WT animals. These effects were accompanied by an attenuation of the TBI-induced deficits in the rotarod and Morris water maze tasks [140]. While ASK1 may be contributing to increased barrier disruption following injury by shifting microglia to a more proinflammatory state, others have observed that microglia can be protective in the early stages of inflammation and later transform to a more reactive profile [63].

In the early stages of systemic inflammation (modeled with daily LPS injection), microglia were recruited to microvessels via EC-secreted CCL5 [63]. TEM revealed that microglia invade the NVU, where they support barrier function. These NVU-invading microglia express CLDN-5 protein, thereby contributing to TJ function. Importantly, the time course of these observations aligned with a decrease in barrier permeability. However, several days into systemic inflammation, microglia began to express phagocytic marker CD68. These phagocytic microglia were observed to have aquaporin-4 inclusions, indicating engulfment of astrocytic end feet. The timing of these observations correlated with an increase in barrier permeability [63]. These findings suggest that microglia engaged by systemic inflammation can be both protective and harmful to barrier function.

Other work has implicated phagocytosis by activated microglia in barrier disruption following middle cerebral artery occlusion (MCAO) [138]. In response to injury, microglia shifted to an activated morphological phenotype and migrated towards blood vessels. Microglia engulfed and phagocytosed blood vessels, leading to increased barrier disruption and extravasation of blood products. CX3CR1 KO animals, with reduced microglial activation and function, showed a significant reduction in stroke size and extravasation of a contrast agent at 24 h post-MCAO compared to WT animals [138]. The time course of these events suggests that microglia migration and engulfment of blood vessels may contribute to increased barrier disruption.

It appears that microglia can have varied effects on BBB integrity. In some scenarios, microglial activation seems necessary, providing surveillance for damage to the nervous system. Microglia activation during early systemic inflammation was shown to be protective against BBB disruption. Over time, the response of microglia changed and negative effects emerged. Microglia were also shown to be activated as a result of neurotrauma in vivo or with exposure to ROS and LPS in vitro. There is a compounding effect where microglia are activated by ROS or proinflammatory cytokines, and this activation results in increased production of ROS and cytokines, thereby fueling a positive feedback loop. The presence of these molecules ultimately promotes unchecked neuroinflammation, which can be deleterious to recovery from injury.

### 6.2. De Novo SUR1-Trpm4 Expression and Progressive Hemorrhagic Necrosis (PHN)

The initial injury in neurotrauma produces some hemorrhage due to shearing and the physical damage to blood vessels. Over the course of the next 24 h, hemorrhage expansion has been observed in both animal models and humans [6]. This change has been attributed to the presence of small microbleeds, called petechial hemorrhages, that appear both proximal and distal to the initial lesion. Both *Abcc8*/SUR1 and Trpm4 have been implicated. The gene *Abcc8* encodes the ATP-binding cassette (ABC) transporter, sulfonylurea receptor 1 (SUR1). Unlike many ABC transporters, SUR1 associates with pore-forming subunits of transient receptor potential melastatin 4 (Trpm4) to create a SUR1-regulated nonselective, calcium-activated, and ATP-sensitive cation channel [6,141]. Activation of the complex, either through ATP depletion or increased levels of intracellular calcium, leads to the opening of the channel, which allows an inward sodium current. This inward current results in depolarization and oncotic cell swelling, which can lead to cell death [5]. This de novo expression of SUR1 was observed in areas surrounding ischemic and contusion lesions in neurons, astrocytes, and endothelial cells [142,143].

Researchers have observed increased SUR1 expression in humans and rodents in the penumbral region after SCI, with a gradual reduction with distance from the lesion site [6]. Genetic silencing of *Abcc8* in mice and pharmacological inhibition of SUR1 in rats (via antisense oligodeoxy-nucleotides or glibenclamide) reduced lesion volume, petechial hemorrhages, and capillary fragmentation. Furthermore, blocking *Abcc8* or SUR1 improved trunk stability and locomotor function [6].

On its own, SUR1 has no known function, whereas Trpm4 alone can form functional channels [5]. Neural injury has been associated with the de novo expression of the SUR1-Trpm4 complex, which is otherwise not constitutively expressed in the CNS. In the setting of hypoxia, hypoxia-inducible factor (Hif) binds to the *Sp1* promoter, resulting in its transcription, which increases expression of *Abcc8*. Cytokine expression may also increase SUR1 expression as TNF-α increases nuclear p65 NF-*κ*B, which can interact with the *Abcc8* promoter to increase *Abcc8* gene expression [5]. Given that hypoxia and cytokine expression are common after neural injury, these processes could fuel SUR1 expression in the CNS.

SUR1 forms from two complexes, one with Kir6.2 and the other with Trpm4 [144]. Following intracerebral hemorrhage, SUR1 expression increased while Kir6.2 did not, suggesting that the SUR1 upregulation was related to the formation of SUR1-Trpm4 complexes. Jiang et al. (2017) showed that SUR1 expression is concentrated in the injured and surrounding tissue [144]. Additionally, they provided evidence of the protective nature of glibenclamide treatment, which reduced intracerebral hemorrhage-induced edema, Evan’s blue extravasation, and motor/learning deficits [144]. Another group showed that KO of Trpm4 in a model of MCAO had a protective effect comparable to that of glibenclamide [141]. When tested separately, glibenclamide, glimepiride (another sulfonylurea similar to glibenclamide but with less risk of hypoglycemia), or KO of Trpm4 reduced neurological and motor deficits, infarct volume, edema formation, and Evan’s blue extravasation after injury [141]. The protective effects of glibenclamide following SCI were found to be limited to a particular injury model [145]. Subsequent work examining the replicability of these results revealed that glibenclamide only reduced the expansion of hemorrhage in more lateral injuries that were angled in relation to the spinal cord axis [145].

We previously discussed how nociceptive sensory activity after a spinal cord injury can promote hemorrhage after SCI [4]. A similar effect has been reported after TBI [113]. In both cases, hemorrhage was accompanied by capillary fragmentation, a hallmark of progressive hemorrhagic necrosis (PHN). In addition, exposure to noxious stimulation after SCI increased the expression of SUR1-Trmp4 at the site of injury, implicating PHN [4].

While most studies examining SUR1-Trpm4 in neural injury have used a preclinical model, human data have also shown SUR1 upregulation post-injury [143]. In tissue from TBI patients, SUR1 was broadly expressed, particularly in neurons and ECs. Over time, expression increased in neurons, glial cells, and macrophages, remained consistent in ECs, and decreased in neutrophils [143]. Though this study was not sufficiently powered to link expression with contusion volume or neurological outcome, it highlights cell-specific SUR1 dynamics in a clinical population. While it is accepted that SUR1-Trpm4 channel engagement is detrimental after injury in preclinical and clinical settings, the protective effects of glibenclamide in human patients have not been demonstrated.

There is ample evidence to implicate SUR1-Trpm4 in the disruption of the BBB/BSCB after neural injury. Furthermore, this complex may be implicated in the increased production of MMPs and ultimately the degradation of TJPs [5,6,141,144,146]. Curbing this process could result in meaningful improvements in clinical outcomes. Further work is warranted to understand how SUR1-Trpm4 complex activity can be modulated to reduce barrier disruption and PHN following neurotrauma.

### 6.3. Peripherally Derived Matrix Metalloproteinases (MMPs) Disrupt the BBB

MMPs are best known for degrading extracellular matrix (ECM) components, a process essential for development and angiogenesis [147,148]. Their roles have expanded with the discovery that MMPs also cleave molecules to produce IL-1, VEGF, and TGF-β. MMP activity is tightly regulated under physiological conditions [147]. While activation typically occurs via proteolytic cleavage, autocleavage can be triggered by ROS, thiol-modifying agents, plasmin, and thrombin [147,149,150]. While endogenous tissue inhibitors of metalloproteinases (TIMPs) primarily inhibit MMP activation, they can also facilitate it, particularly in the case of MMP-2 [147]. MMPs are heavily implicated in neural injury [148]. The initial barrier breakdown leads to the infiltration of neutrophils and an immune response from resident cells, increasing production of MMPs, particularly MMP-2 and MMP-9. While secreted by many cell types within the CNS, including astrocytes and ECs, neutrophils are a primary source of MMPs following neural injury [44,51,148,151,152]. Furthermore, several TJPs, such as OCLN, CLDN, and ZO-1, are degraded by MMPs [148,153,154,155].

Haorah et al. (2007) demonstrated that ROS-induced activation of MMPs is mediated by protein tyrosine kinase (PTK) in human brain microvascular ECs [151]. ROS increased MMP activity and ECM degradation, while TIMP suppression enhanced PTK activity and triggered tyrosine phosphorylation of OCLN, CLDN-5, and ZO-1. In addition, TEER was reduced, and this was associated with increased barrier permeability and monocyte migration. All of these effects were largely reversed by genistein, a PTK inhibitor, highlighting PTK’s role in ROS-driven MMP activation and barrier disruption.

Others have linked receptor tyrosine kinase activation to MAPK signaling and increased activity. Specifically, ERK activation is associated with TJP disruption and increased barrier permeability [156,157]. In vitro, TNF-α induced MMP-9 expression in brain microvascular ECs, disrupting ZO-1 integrity. These effects were blocked by inhibiting MMP-9, TNF receptors, NF-kB, or MAPK pathways (ERK 1/2, p38, and JNK 1/2) [157]. Other studies implicated Ca^2+^CaMKII/ERK/NF-kB signaling in TNF-α-mediated MMP-9 upregulation [156]. Here, TNF-α also reduced TEER, increased MMP-9 and NF-kB activity, and decreased cytoplasmic NF-kB. These effects were attenuated by propofol, calcium blockade, or silencing MMP-2/MMP-9. Together, these findings suggest that TNF-α activates MAPK and NF-kB pathways via ERK signaling, promoting MMP-9 expression and barrier disruption.

ERK signaling has been implicated in the upregulation of MMPs, inflammation, and hypoxia. In mouse models of TBI, ERK phosphorylation increased within minutes, followed by elevated MMP-9 expression, ZO-1 disruption, and cerebral edema at 24 h. These effects were reduced by U0126, an ERK inhibitor, which also decreased lesion size at one week [154]. Similarly, in the MCAO rat model, U0126 reduced lesion volume, neurological deficits, and injury-induced increases in MMP-9 and TIMP-1 when administered after reperfusion [158]. Other in vivo studies have shown that hypoxic exposure increases barrier permeability and disrupts ZO-1 and occludin organization, changes associated with MMP-9 upregulation. These effects were mitigated by MMP inhibition, supporting a role for MMPs in TJ disruption and barrier dysfunction after neural injury [153]. Together, these findings demonstrate that upregulation of MMPs via ERK activation results in the degradation of TJP complexes and functional disruption of the CNS barrier [153,154,156,157,158].

Under physiological conditions, MMPs are needed for a number of processes, including the maintenance of the ECM. In the setting of neural injury, MMP production is increased, and infiltrating immune cells fuel this process. This can trigger a hypoxic environment and the expression of proinflammatory cytokines, a characteristic of many CNS pathologies. This, in turn, engages PTK and various MAPK pathways that result in the activation of NF-kB, which can increase transcription of MMP genes. Treatments that reduce MMPs may protect the BBB and improve neurological outcomes following CNS injuries.

## 7. Conclusions and Further Considerations

The preclinical studies reviewed above detail the factors that influence BBB/BSCB function and the underlying mechanisms, setting the stage for clinical translation. Below, we summarize current clinical studies, discuss future directions, and provide a summary of the processes that contribute to barrier disruption after neural injury.

### 7.1. Clinical Therapeutics

Currently, clinical treatment for many neurological injuries focuses on minimizing secondary damage, such as reperfusion after stroke and decompression after TBI or SCI. While it is clear that repairing and minimizing barrier damage may be helpful, the effects of conventional clinical treatments on barrier permeability and TJP levels are not routinely assessed. Emerging research and therapeutics bring increased focus on barrier preservation and repair.

Given the deleterious effects of pain on barrier function, particularly in the context of neurological injury (TBI and SCI), conventional pain management strategies may have therapeutic value [3,4,105,111,113]. Nerve blocks using anesthetics such as bupivacaine or lidocaine have been shown to reduce barrier disruption in preclinical pain models and could be easily translated to treatment in neurological trauma [105,107]. Other work suggests that general anesthesia may be beneficial in cases of neurological injuries with polytrauma [3]. Further work is warranted to assess whether general anesthesia or increased sedation in patients with neurological injuries limits barrier disruption and secondary tissue loss.

While management of hemorrhage and thrombosis after neurological injuries is important to prevent lesion expansion and ischemia, respectively, there is evidence that molecules that mediate fibrinolysis and coagulopathy can affect barrier function [48,62,129,130,159]. Tranexamic acid inhibits fibrinolysis and has been used clinically to control hemorrhage and lesion expansion in TBI [125]. Interestingly, tranexamic acid has also been shown to block tPA-induced increases in barrier permeability in vitro [159], suggesting that it could limit barrier breakdown in other injuries as well. However, it is important to note that the use of tranexamic acid may not be safe in all patients, and the time course of the trauma should be carefully considered [125,126]. Because it inhibits fibrinolysis, there is an increased risk of thrombosis, and many only recommend its use in the early subacute phase of injury.

Sulfonylurea drugs, such as glibenclamide and glyburide, have been shown to block the SUR1-Trpm4 channel and reduce injury severity and deficits in animal models of stroke [141,142,146], TBI [144], and SCI [145]. Glibenclamide has been tested in a placebo-controlled, randomized, clinical trial for the treatment of large hemispheric infarctions [160]. While this trial showed that a 72 h infusion of 8.6 mg of glibenclamide was feasible, there was no improvement in functional outcomes at 90 days post-injury [160]. Another sulfonylurea, glyburide, showed a reduction in vasogenic but not cytotoxic edema in patients receiving intravenous glyburide [161]. It is important to note, however, that these findings are from the retrospective analysis of a pilot study that included just 10 patients presenting with large acute ischemic stroke and treated with open-label glyburide [162]. For the retrospective analysis, glyburide-treated patients were compared to placebo-treated patients in a normobaric oxygen therapy trial for acute stroke [161]. Glyburide has also been evaluated in patients with traumatic cervical spinal cord injury, but was terminated due to low enrollment and logistical issues [163]. A total of two patients were enrolled after switching the route from intravenous to oral and increasing the time allowed from injury to drug administration up to 8 h [163]. A new clinical trial is being implemented to assess the safety and efficacy of oral glyburide in patients with cervical or thoracic spinal cord injuries (NCT05426681). While sulfonylurea drugs may be beneficial after neurological injury, given the preclinical data, more clinical studies are needed to determine their positive effects, if any, in clinical populations.

Another avenue of clinical research is exploring the usefulness of barrier permeability as a diagnostic and prognostic marker. MRI has been used to evaluate barrier disruption and its association with recovery, comorbidities, and overall prognosis [164,165,166]. Barrier disruption has been shown to predict the progression of cerebral small vessel disease following stroke [164]. Research suggests that diffuse damage to the barrier is reversible, while intense focal damage may lead to hemorrhagic transformation in ischemic stroke [165]. The acute extent of barrier disruption predicts recovery in the months following stroke, showing that increased disruption is associated with increased disability [166]. In addition to imaging, biomarkers of barrier disruption, such as serum levels of S100β, may be used for diagnostic and prognostic assessments [69,167,168,169]. Both imaging and biomarker analysis have been used to demonstrate a strong association between barrier disruption and the emergence of cognitive decline [167], Parkinson’s disease [170], and amyotrophic lateral sclerosis [171].

Other work is exploring methods to foster drug delivery across the BBB. Techniques include the use of nanoparticles, intranasal administration, temporary BBB disruption via focused ultrasound, and receptor-mediated transport (for further details, see [172]). Barrier disruption has been used to enhance the delivery of pharmacological treatment for ischemic stroke and reperfusion injury [173]. Tannic acid, polydopamine, and Mo-based heteropolyacid ternary composite nanomedicine (TPM) have been used because these agents bind with collagen, a component of the basal lamina in the BBB, over glycocalyx, which is present in higher levels in intact versus damaged blood vessels. This binding preference allows TPM to enter the disrupted BBB and target mitochondria by binding to the mitochondrial outer membrane protein TOM20. TPM treatment attenuated ischemic stroke-induced infarct area and improved neurologic function by reducing mitochondrial ROS release. This decreased cytochrome c-induced neuronal death and limited the release of mitochondrial DNA, thereby disrupting activation of the STING pathway and the consequential induction of a proinflammatory state in microglia. This reduced the proinflammatory storm that follows stroke and showed that barrier disruption can be used to foster drug delivery [173].

Clinicians are also exploring treatments to rescue and aid in barrier function in human pathologies. For example, adeno-associated viruses (AAVs) can be used to target ECs [174]. This technique has been used to agonize the non-canonical Wnt7a receptor, Gpr124 [175]. This approach was applied to a stroke model, engaging development processes to restore the physiological functioning of the BBB [175]. Likewise, genetic manipulations targeting ECs may be used to deliver gene therapies that protect and even repair TJs, and thereby repair the BBB/BSCB. For example, evidence suggests that AAVs can be used to overexpress CLDN-5 TJP and restore barrier function in vivo [176]. Further work is needed to evaluate the therapeutic value of treatments identified through preclinical studies.

### 7.2. Future Directions

**Figure 7:**
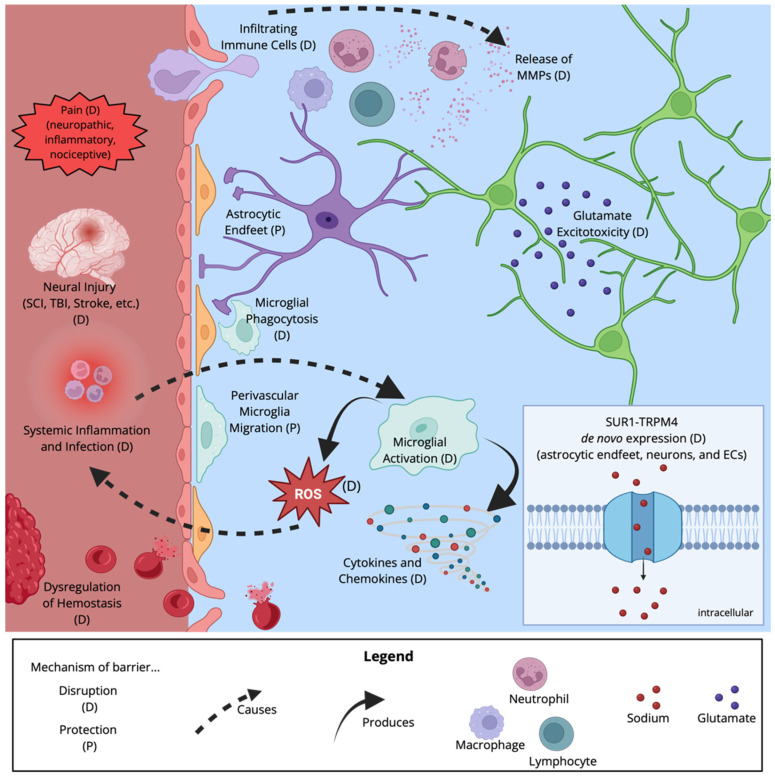
Mechanisms of BBB/BSCB disruption and protection. Key components involve different types of pain, neurological injuries, systemic inflammation, dysregulation of hemostasis, immune cells infiltrating the CNS, release of MMPs, glutamate excitotoxicity, microglia migration and phagocytosis, astrocytic end feet, release of cytokines and chemokines, and SUR-1 TRPM4 expression. Many of these mechanisms disrupt (D) the barrier, while a few protect (P) it. Created with Biorender.com.

It is clear that the BBB and BSCB are crucial to the healthy physiological functioning of the CNS and that many factors can modulate these barriers. In neural injury, the barriers are disrupted by physical and mechanical forces. This initial injury allows for the entrance of peripheral substances that drive neuroinflammation, resulting in an inflammatory response that further degrades the barrier. This vicious cycle is implicated in the negative outcomes following neurotrauma, making prevention of the secondary barrier disruption a key therapeutic target. In particular, secondary injury brings increased proinflammatory cytokines, MMPs, oxidative stress, immune cell infiltration, excitotoxicity, and changes in coagulopathy, all of which disrupt TJPs. These TJPs, which form the glue that maintains the BBB/BSCB, respond with alterations in structural organization and expression of mRNA and protein. Figure 7 illustrates the mechanisms contributing to alterations in barrier permeability.

**Figure 8:**
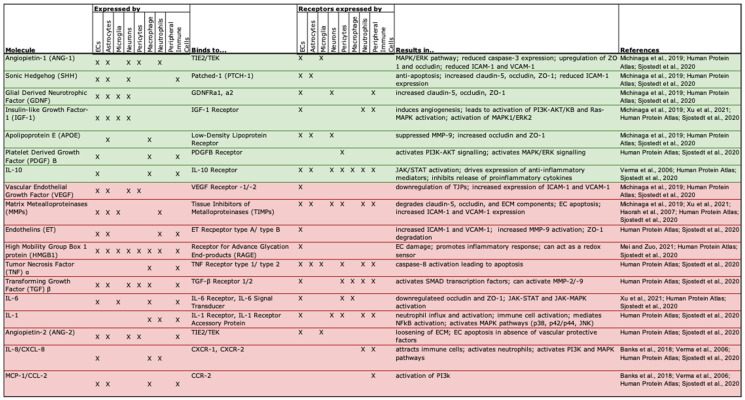
Prominent signaling molecules affecting the BBB/BSCB. The Table lists key molecules, their receptor(s), and the cell types that express them. The letter X is used to indicate cells types expressing the molecules and their corresponding receptors. Rows in green indicate factors that a protective to the barriers while those in red are generally disruptive. Michinaga et al., 2019 [44]; Human Protein Atlas [177]; Sjostedt et al., 2020 [178]; Xu et al., 2021 [51]; Verma et al., 2006 [29]; Haorah et al., 2007 [151]; Mei and Zuo, 2021 [121]; Banks et al., 2018 [28].

Many prominent molecules involved in barrier disruption, such as inflammatory cytokines and chemokines, are secreted from more than one cell type in the CNS. Furthermore, receptors that interact with these molecules are often displayed on more than one cell type as well, a complexity that must be considered when determining how activation of a cell type or release of a molecule affects the BBB/BSCB. This is also relevant to the development of therapeutics because there may be unanticipated effects of any treatment. Lack of acknowledgement of potential off-target effects contributes to the failed identification and translation of novel therapeutics. Figure 8 provides an overview of prominent signaling molecules and cell types implicated.

While it is clear that TJP disruption is implicated in increased barrier permeability, it is likely that preventing this process will at most limit tissue loss. The initial injury causes hemorrhage, which expands over the coming days and weeks [6]. Mitigating disruption to TJs, or restoring their structure, could limit hemorrhage spread in the acute phase. Furthermore, less severe injuries may benefit from early treatment.

While limiting the extent of the secondary injury following neurotrauma is of critical importance, the disruption of TJs has other implications. Following neural injury, “leaky barriers” have been reported in the lungs and gastrointestinal tract [118,179,180,181]. Several groups have shown that factors such as pain and systemic inflammation can broadly disrupt barrier integrity. Beggs et al. (2010) showed a gradient of disruption to the CNS barrier and an increase in peripheral endothelial barrier permeability using an inflammatory paw pain model [107]. Others have shown that lung infection months after a TBI resulted in increased neuroinflammation and a local inflammatory response within the lungs [118]. Limiting the disruption of TJs could reduce the extent of barrier disruption throughout the body.

As noted above, neural injury causes physical damage to the BBB/BSCB, allowing entry of harmful substances. Furthermore, the inflammatory response to injury creates toxic products that can negatively affect cellular function, fueling cell death, which adds to the waste load. The impairment of waste clearance could contribute to the high prevalence of neurodegenerative disease in patients following TBI [62,182,183]. Neural injury-induced functional disruptions could chronically increase barrier permeability, resulting in long-term sequelae. Preventing this disruption may provide long-term benefits beyond the acute period. While BBB/BSCB disruption has not been shown as an explicit cause, it is closely associated with many neurodegenerative disorders. Chronic barrier disruption may lead to an extended neuroinflammatory response and thereby predispose the CNS to neurodegeneration.

There is also ample work relating BBB disruption to long-term neuroinflammation. BBB disruption has been implicated in epilepsy, a common symptom following TBI, which has parallels to spasticity after SCI. Both epilepsy and spasticity are linked to aberrant spontaneous neuronal activity and hyperexcitability within the CNS. Chronic pain is another common symptom observed in neurotrauma survivors. The link between neuroinflammation and the development of chronic pain has been proposed before [184,185]. Limiting barrier breakdown could reduce neuroinflammation and the infiltration of peripheral immune cells, and thereby mitigate the development of these effects.

### 7.3. Summary

The studies reviewed here have highlighted the critical role of the BBB/BSCB as well as contributions from various cells of the NVU and TJPs. The barrier forms early in development and is closely coupled with angiogenesis. Although crucial to homeostatic functioning of the CNS, the barrier undergoes changes due to aging that result in increased permeability.

A multitude of both peripheral and central factors can alter the barrier and its functions. Nociceptive signaling results in a disruption to both central and peripheral barriers. This effect is blocked by inhibiting signal transduction or eliminating pain processing in the brain. Systemic inflammation can also disrupt the barrier. Importantly, there is ample evidence for a bidirectional relationship between systemic inflammation and neuroinflammation. While it is alarming that inflammation in either system can spread to the other, this also suggests that management of one may be protective for both. Deviations from homeostatic coagulation and fibrinolysis are also common after injury and could disrupt barrier function through the many products and signaling molecules of their respective cascades.

While changes outside of the nervous system clearly produce disruption of the barrier, changes within do as well. Microglia, as resident immune cells of the CNS, can become activated and have been shown to have both protective and deleterious functions. Activated microglia produce cytokines and ROS, as well as phagocytose waste and, in some cases, astrocytic end feet. PHN, as a result of the de novo synthesis of the SUR1-Trpm4 complex, results in the expansion of the lesion over the acute period in the form of petechial hemorrhages.

Although reversal of the initial damage to the CNS is not possible, damage could be limited by mitigating the expansion of hemorrhage. MMPs have been implicated and lead to the disassembly of TJPs, resulting in disruption to the BBB/BSCB. MMPs are produced by many cell types, but this is exacerbated with the infiltration of neutrophils following neurotrauma.

Many of the processes and events resulting in barrier disruption overlap. This intertwinement adds to the complexity of elucidating the causal relationship and must be considered when developing therapeutics. Targeting barrier function following injury will not cure SCI or TBI. It may, however, lead to meaningful improvements in clinical management.

## Data Availability

No new data were created or analyzed in this study.

## Abbreviations

The following abbreviations are used in this manuscript:

BBB	Blood–brain barrier
BSCB	Blood–spinal cord barrier
CNS	Central nervous system
SCI	Spinal cord injury
TBI	Traumatic brain injury
EC(s)	Endothelial cell(s)
TJ(s)	Tight junction(s)
TJP(s)	Tight junction protein(s)
NVU	Neurovascular unit
CSF	Cerebrospinal fluid
IPAD	Intramural periarterial drainage
VEGF	Vascular endothelial growth factor
PDGF	Platelet-derived growth factor
Sp	Streptococcus Pneumoniae
tPA	Tissue-type plasminogen activator
ICAM	Intercellular adhesion molecule
VCAM	Vascular cell adhesion molecule
IGF2	Insulin-like growth factor 2
KO	Knock out
MMP	Matrix metalloproteinase
ROS	Reactive oxygen species
TEER	Transendothelial electrical resistance
DCE-MRI	Dynamic contrast enhanced-magnetic resonance imaging
PET	Positron emission tomography
SPECT	Single-photon emission computed tomography
TAMP(s)	Tight-junction-associated MARVEL proteins
ZO(s)	Zonula occludens
ECL(s)	Extracellular loops
JAM(s)	Junctional adhesion molecules
MARVEL	[Myeloid and lymphocyte] and related proteins for vesicle trafficking
TEM	Transmission electron microscopy
CFA	Complete Freund’s adjuvant
LPS	Lipopolysaccharide
AOPP(s)	Advanced oxidation protein products
MCAO	Middle cerebral artery occlusion
PHN	Progressive hemorrhagic necrosis
ABC	ATP-binding cassette
SUR1	Sulfonylurea receptor 1
Trpm4	Transient receptor potential melastatin 4
Hif	Hypoxia-inducible factor
ECM	Extracellular matrix
TIMP(s)	Tissue inhibitors of metalloproteinases
PTK	Protein tyrosine kinase
TPM	Ternary composite nanomedicine
AAV(s)	Adeno-associated viruses

## References

[1] Dotiwala A.K., McCausland C., Samra N.S. (2023). Anatomy, Head and Neck: Blood Brain Barrier. Stats Pearl.

[2] Beattie M.S., Hermann G.E., Rogers R.C., Bresnahan J.C., McKerracher L., Doucet G., Rossignol S. (2002). Cell Death in Models of Spinal Cord Injury. Progress in Brain Research.

[3] Davis J.A., Bopp A.C., Henwood M.K., Bean P., Grau J.W. (2023). General anesthesia blocks pain-induced hemorrhage and locomotor deficits after spinal cord injury in rats. J. Neurotrauma.

[4] Turtle J.D., Henwood M.K., Strain M.M., Huang Y.-J., Miranda R.C., Grau J.W. (2019). Engaging pain fibers after a spinal cord injury fosters hemorrhage and expands the area of secondary injury. Exp. Neurol..

[5] Jha R.M., Rani A., Desai S.M., Raikwar S., Mihaljevic S., Munoz-Casabella A., Kochanek P.M., Catapano J., Winkler E., Citerio G. (2021). Sulfonylurea receptor 1 in central nervous system injury: An updated review. Int. J. Mol. Sci..

[6] Simard J.M., Woo S.K., Norenberg M.D., Tosun C., Chen Z., Ivanova S., Tsymbalyuk O., Bryan J., Landsman D., Gerzanich V. (2010). Brief suppression of Abcc8 prevents autodestruction of spinal cord after trauma. Sci. Transl. Med..

[7] van Vliet E.A., da Costa Araujo S., Redeker S., van Schaik R., Aronica E., Gorter J.A. (2007). Blood-brain barrier leakage may lead to progression of temporal lobe epilepsy. Brain.

[8] Marchi N., Granata T., Ghosh C., Janigro D. (2012). Blood-brain barrier dysfunction and epilepsy: Pathophysiologic role and therapeutic approaches. Epilepsia.

[9] Yang C., Hawkins K.E., Dore S., Candelario-Jalil E. (2019). Neuroinflammatory mechanisms of blood-brain barrier damage in ischemic stroke. Am. J. Physiol. Cell Physiol..

[10] Cai Z., Qiao P.F., Wan C.Q., Cai M., Zhou N.K., Li Q. (2018). Role of Blood-Brain Barrier in Alzheimer’s Disease. J. Alzheimers Dis..

[11] Galea I. (2021). The blood-brain barrier in systemic infection and inflammation. Cell. Mol. Immunol..

[12] Dal-Pizzol F., Rojas H.A., dos Santos E.M., Vuolo F., Constantino L., Feier G., Pasquali M., Comim C.M., Petronilho F., Gelain D.P. (2013). Matrix metalloproteinase-2 and metalloproteinase-9 activities are associated with blood-brain barrier dysfunction in an animal model of severe sepsis. Mol. Neurobiol..

[13] Atis M., Akcan U., Ugur Yilmaz C., Orhan N., Duzgun P., Deniz Ceylan U., Arican N., Karahuseyinoglu S., Nur Sahin G., Ahishali B. (2019). Effects of methyl-beta-cyclodextrin on blood-brain barrier permeability in angiotensin II-induced hypertensive rats. Brain Res..

[14] Stamatovic S.M., Johnson A.M., Keep R.F., Andjelkovic A.V. (2016). Junctional proteins of the blood-brain barrier: New insights into function and dysfunction. Tissue Barriers.

[15] Tietz S., Engelhardt B. (2015). Brain barriers: Crosstalk between complex tight junctions and adherens junctions. J. Cell Biol..

[16] Bartanusz V., Jezova D., Alajajian B., Digicaylioglu M. (2011). The blood-spinal cord barrier: Morphology and clinical implications. Ann. Neurol..

[17] Prockop L.D., Naidu K.A., Binard J.E., Ransohoff J. (1995). Selective permeability of [3H]-D-mannitol and [14C]-carboxyl-inulin across the blood-brain barrier and blood-spinal cord barrier in the rabbit. J. Spinal Cord. Med..

[18] Pan W., Banks W.A., Kastin A.J. (1997). Permeability of the blood–brain and blood–spinal cord barriers to interferons. J. Neuroimmunol..

[19] Ge S., Pachter J.S. (2006). Isolation and culture of microvascular endothelial cells from murine spinal cord. J. Neuroimmunol..

[20] Brown L.S., Foster C.G., Courtney J.M., King N.E., Howells D.W., Sutherland B.A. (2019). Pericytes and Neurovascular Function in the Healthy and Diseased Brain. Front. Cell. Neurosci..

[21] Thomsen M.S., Routhe L.J., Moos T. (2017). The vascular basement membrane in the healthy and pathological brain. J. Cereb. Blood Flow. Metab..

[22] Iadecola C. (2017). The Neurovascular Unit Coming of Age: A Journey through Neurovascular Coupling in Health and Disease. Neuron.

[23] Andjelkovic A.V., Situ M., Citalan-Madrid A.F., Stamatovic S.M., Xiang J., Keep R.F. (2023). Blood-Brain Barrier Dysfunction in Normal Aging and Neurodegeneration: Mechanisms, Impact, and Treatments. Stroke.

[24] Franzen O., Gan L.M., Bjorkegren J.L.M. (2019). PanglaoDB: A web server for exploration of mouse and human single-cell RNA sequencing data. Database.

[25] Tarhan L., Bistline J., Chang J., Galloway B., Hanna E., Weitz E. (2023). Single Cell Portal: An interactive home for single-cell genomics data. bioRxiv.

[26] Banks W.A. (2012). Brain meets body: The blood-brain barrier as an endocrine interface. Endocrinology.

[27] Banks W.A. (2019). The blood-brain barrier as an endocrine tissue. Nat. Rev. Endocrinol..

[28] Banks W.A., Kovac A., Morofuji Y. (2018). Neurovascular unit crosstalk: Pericytes and astrocytes modify cytokine secretion patterns of brain endothelial cells. J. Cereb. Blood Flow. Metab..

[29] Verma S., Nakaoke R., Dohgu S., Banks W.A. (2006). Release of cytokines by brain endothelial cells: A polarized response to lipopolysaccharide. Brain Behav. Immun..

[30] Thornton P., McColl B.W., Greenhalgh A., Denes A., Allan S.M., Rothwell N.J. (2010). Platelet interleukin-1alpha drives cerebrovascular inflammation. Blood.

[31] Gidday J.M., Gasche Y.G., Copin J.-C., Shah A.R., Perez R.S., Shapiro S.D., Chan P.H., Park T.S. (2005). Leukocyte-derived matrix metalloproteinase-9 mediates blood-brain barrier breakdown and is proinflammatory after transient focal cerebral ischemia. Am. J. Physiol. Heart Circ. Physiol..

[32] Zhu Y., Xu J., Chai Y., Li P., Liu L., Zhang S., Zhang J., Chen X. (2025). Neutrophil extracellular traps aggravate blood-brain barrier disruption via ZBP1/FSP1-mediated ferroptosis after traumatic brain injury. Fluids Barriers CNS.

[33] Furlan R., Johnson H.L., Willenbring R.C., Jin F., Manhart W.A., LaFrance S.J., Pirko I., Johnson A.J. (2014). Perforin Competent CD8 T Cells Are Sufficient to Cause Immune-Mediated Blood-Brain Barrier Disruption. PLoS ONE.

[34] Kadry H., Noorani B., Cucullo L. (2020). A blood-brain barrier overview on structure, function, impairment, and biomarkers of integrity. Fluids Barriers CNS.

[35] Li P., Fan H. (2023). Pericyte Loss in Diseases. Cells.

[36] Attwell D., Mishra A., Hall C.N., O’Farrell F.M., Dalkara T. (2016). What is a pericyte?. J. Cereb. Blood Flow. Metab..

[37] Hariharan A., Weir N., Robertson C., He L., Betsholtz C., Longden T.A. (2020). The Ion Channel and GPCR Toolkit of Brain Capillary Pericytes. Front. Cell. Neurosci..

[38] Bell R.D., Winkler E.A., Sagare A.P., Singh I., LaRue B., Deane R., Zlokovic B.V. (2010). Pericytes control key neurovascular functions and neuronal phenotype in the adult brain and during brain aging. Neuron.

[39] Hall C.N., Reynell C., Gesslein B., Hamilton N.B., Mishra A., Sutherland B.A., O’Farrell F.M., Buchan A.M., Lauritzen M., Attwell D. (2014). Capillary pericytes regulate cerebral blood flow in health and disease. Nature.

[40] Hariharan A., Robertson C.D., Garcia D.C.G., Longden T.A. (2022). Brain capillary pericytes are metabolic sentinels that control blood flow through a K(ATP) channel-dependent energy switch. Cell Rep..

[41] Pandey K., Bessieres B., Sheng S.L., Taranda J., Osten P., Sandovici I., Constancia M., Alberini C.M. (2023). Neuronal activity drives IGF2 expression from pericytes to form long-term memory. Neuron.

[42] Armulik A., Genove G., Mae M., Nisancioglu M.H., Wallgard E., Niaudet C., He L., Norlin J., Lindblom P., Strittmatter K. (2010). Pericytes regulate the blood-brain barrier. Nature.

[43] Daneman R., Zhou L., Kebede A.A., Barres B.A. (2010). Pericytes are required for blood-brain barrier integrity during embryogenesis. Nature.

[44] Michinaga S., Koyama Y. (2019). Dual Roles of Astrocyte-Derived Factors in Regulation of Blood-Brain Barrier Function after Brain Damage. Int. J. Mol. Sci..

[45] Sofroniew M.V., Vinters H.V. (2010). Astrocytes: Biology and pathology. Acta Neuropathol..

[46] Zhou R., Li J., Wang R., Chen Z., Zhou F. (2023). The neurovascular unit in healthy and injured spinal cord. J. Cereb. Blood Flow. Metab..

[47] Hasel P., Rose I.V.L., Sadick J.S., Kim R.D., Liddelow S.A. (2021). Neuroinflammatory astrocyte subtypes in the mouse brain. Nat. Neurosci..

[48] Briens A., Bardou I., Lebas H., Miles L.A., Parmer R.J., Vivien D., Docagne F. (2017). Astrocytes regulate the balance between plasminogen activation and plasmin clearance via cell-surface actin. Cell Discov..

[49] Barker-Haliski M., White H.S. (2015). Glutamatergic mechanisms associated with seizures and epilepsy. Cold Spring Harb. Perspect. Med..

[50] Rothstein J.D., Dykes-Hoberg M., Pardo C.A., Bristol L.A., Jin L., Kuncl R.W., Kanai Y., Hediger M.A., Wang Y., Schielke J.P. (1996). Knockout of glumate transporters reveals a major role for astroglial transport in excitotoxicity and clearance of glutamate. Neuron.

[51] Xu L., Wang J., Ding Y., Wang L., Zhu Y.J. (2021). Current Knowledge of Microglia in Traumatic Spinal Cord Injury. Front. Neurol..

[52] da Fonseca A.C., Matias D., Garcia C., Amaral R., Geraldo L.H., Freitas C., Lima F.R. (2014). The impact of microglial activation on blood-brain barrier in brain diseases. Front. Cell. Neurosci..

[53] Hammond T.R., Dufort C., Dissing-Olesen L., Giera S., Young A., Wysoker A., Walker A.J., Gergits F., Segel M., Nemesh J. (2019). Single-Cell RNA Sequencing of Microglia throughout the Mouse Lifespan and in the Injured Brain Reveals Complex Cell-State Changes. Immunity.

[54] Takata F., Nakagawa S., Matsumoto J., Dohgu S. (2021). Blood-Brain Barrier Dysfunction Amplifies the Development of Neuroinflammation: Understanding of Cellular Events in Brain Microvascular Endothelial Cells for Prevention and Treatment of BBB Dysfunction. Front. Cell. Neurosci..

[55] Crouch E.E., Joseph T., Marsan E., Huang E.J. (2023). Disentangling brain vasculature in neurogenesis and neurodegeneration using single-cell transcriptomics. Trends Neurosci..

[56] Langen U.H., Ayloo S., Gu C. (2019). Development and Cell Biology of the Blood-Brain Barrier. Annu. Rev. Cell Dev. Biol..

[57] Stewart P.A., Wiley M.J. (1981). Developing nervous tissue induces formation of blood-brain barrier characteristics in invading endothelial cells: A study using quail-chick transplantation chimeras. Dev. Biol..

[58] Vazana U., Veksler R., Pell G.S., Prager O., Fassler M., Chassidim Y., Roth Y., Shahar H., Zangen A., Raccah R. (2016). Glutamate-Mediated Blood-Brain Barrier Opening: Implications for Neuroprotection and Drug Delivery. J. Neurosci..

[59] Cong X., Kong W. (2020). Endothelial tight junctions and their regulatory signaling pathways in vascular homeostasis and disease. Cell. Signal..

[60] Lochhead J.J., Yang J., Ronaldson P.T., Davis T.P. (2020). Structure, Function, and Regulation of the Blood-Brain Barrier Tight Junction in Central Nervous System Disorders. Front. Physiol..

[61] Yang A.C., Stevens M.Y., Chen M.B., Lee D.P., Stahli D., Gate D., Contrepois K., Chen W., Iram T., Zhang L. (2020). Physiological blood-brain transport is impaired with age by a shift in transcytosis. Nature.

[62] Kaur J., Tuor U.I., Zhao Z., Barber P.A. (2011). Quantitative MRI reveals the elderly ischemic brain is susceptible to increased early blood-brain barrier permeability following tissue plasminogen activator related to claudin 5 and occludin disassembly. J. Cereb. Blood Flow. Metab..

[63] Haruwaka K., Ikegami A., Tachibana Y., Ohno N., Konishi H., Hashimoto A., Matsumoto M., Kato D., Ono R., Kiyama H. (2019). Dual microglia effects on blood brain barrier permeability induced by systemic inflammation. Nat. Commun..

[64] Lee S., Kang B.M., Kim J.H., Min J., Kim H.S., Ryu H., Park H., Bae S., Oh D., Choi M. (2018). Real-time in vivo two-photon imaging study reveals decreased cerebro-vascular volume and increased blood-brain barrier permeability in chronically stressed mice. Sci. Rep..

[65] Lindenau K.L., Barr J.L., Higgins C.R., Sporici K.T., Brailoiu E., Brailoiu G.C. (2022). Blood-Brain Barrier Disruption Mediated by FFA1 Receptor-Evidence Using Miniscope. Int. J. Mol. Sci..

[66] Harris W.J., Asselin M.C., Hinz R., Parkes L.M., Allan S., Schiessl I., Boutin H., Dickie B. (2023). In vivo methods for imaging blood-brain barrier function and dysfunction. Eur. J. Nucl. Med. Mol. Imaging.

[67] Milej D., Abdalmalak A., Desjardins L., Ahmed H., Lee T.Y., Diop M., Lawrence K.S. (2017). Quantification of blood-brain barrier permeability by dynamic contrast-enhanced NIRS. Sci. Rep..

[68] Marchi N., Fazio V., Cucullo L., Kight K., Masaryk T., Barnett G., Volgelbaum M., Kinter M., Rasmussen P., Mayberg M.R. (2003). Serum transthyretin monomer as a possible marker of blood-to-CSF barrier disruption. J. Neurosci..

[69] Marchi N., Rasmussen P., Kapural M., Fazio V., Kight K., Mayberg M.R., Kanner A., Ayumar B., Albensi B., Cavaglia M. (2003). Peripheral markers of brain damage and blood-brain barrier dysfunction. Restor. Neurol. Neurosci..

[70] Luissint A.-C., Artus C.d., Glacial F., Ganeshamoorthy K., Couraud P.-O. (2012). Tight junctions at the blood brain barrier: Physiological architecture and disease-associated dysregulation. Fluids Barriers CNS.

[71] Gonzalez-Mariscal L., Betanzos A., Nava P., Jaramillo B.E. (2003). Tight junction proteins. Prog. Biophys. Mol. Biol..

[72] Tornavaca O., Chia M., Dufton N., Almagro L.O., Conway D.E., Randi A.M., Schwartz M.A., Matter K., Balda M.S. (2015). ZO-1 controls endothelial adherens junctions, cell-cell tension, angiogenesis, and barrier formation. J. Cell Biol..

[73] Brooks T.A., Hawkins B.T., Huber J.D., Egleton R.D., Davis T.P. (2005). Chronic inflammatory pain leads to increased blood-brain barrier permeability and tight junction protein alterations. Am. J. Physiol. Heart Circ. Physiol..

[74] Denninger A.R., Breglio A., Maheras K.J., LeDuc G., Cristiglio V., Deme B., Gow A., Kirschner D.A. (2015). Claudin-11 Tight Junctions in Myelin Are a Barrier to Diffusion and Lack Strong Adhesive Properties. Biophys. J..

[75] Kakogiannos N., Ferrari L., Giampietro C., Scalise A.A., Maderna C., Rava M., Taddei A., Lampugnani M.G., Pisati F., Malinverno M. (2020). JAM-A Acts via C/EBP-alpha to Promote Claudin-5 Expression and Enhance Endothelial Barrier Function. Circ. Res..

[76] Martin-Padura I., Lostaglio S., Schneemann M., Williams L., Romano M., Fruscella P., Panzeri C., Stoppacciaro A., Ruco L., Villa A. (1998). Junctional adhesion molecule, a novel member of the immunoglobulin superfamily that distributes at intercellular junctions and modulates monocytes transmigration. J. Cell Biol..

[77] Ronaldson P.T., Davis T.P. (2011). Targeting blood-brain barrier changes during inflammatory pain: An opportunity for optimizing CNS drug delivery. Ther. Deliv..

[78] Morita K., Furuse M., Fujimoto K., Tsukita S. (1999). Claudin multigene family encoding four-transmembrane domain protein components of tight junction strands. Proc. Natl. Acad. Sci. USA.

[79] Tsukita S., Furuse M. (2000). Pores in the Wall: Claudins constitute tight junction strands containing aqueous pores. J. Cell Biol..

[80] Greene C., Hanley N., Campbell M. (2019). Claudin-5: Gatekeeper of neurological function. Fluids Barriers CNS.

[81] Van Itallie C., Rahner C., Anderson J.M. (2001). Regulated expression of claudin-4 decreases paracellular conductance through a selective decrease in sodium permeability. J. Clin. Investig..

[82] Pouyiourou I., Fromm A., Piontek J., Rosenthal R., Furuse M., Gunzel D. (2025). Ion permeability profiles of renal paracellular channel-forming claudins. Acta Physiol..

[83] Simon D.B., Lu Y., Choate K.A., Velazquez H., Al-Sabban E., Praga M., Casari G., Bettinelli A., Colussi G., Rodriguez-Soriano J. (1999). Paracellin-1, a renal tight junction protein required for paracellular Mg2+ resorption. Science.

[84] Raleigh D.R., Marchiando A.M., Zhang Y., Shen L., Sasaki H., Wang Y., Long M., Turner J.R. (2010). Tight Junction-associated MARVEL proteins marvelD3, tricellulin, and occludin have distinct but overlapping functions. Mol. Biol. Cell.

[85] Haseloff R.F., Dithmer S., Winkler L., Wolburg H., Blasig I.E. (2015). Transmembrane proteins of the tight junctions at the blood-brain barrier: Structural and functional aspects. Semin. Cell Dev. Biol..

[86] Van Itallie C.M., Fanning A.S., Holmes J., Anderson J.M. (2010). Occludin is required for cytokine-induced regulation of tight junction barriers. J. Cell Sci..

[87] Hoffman W.H., Stamatovic S.M., Andjelkovic A.V. (2009). Inflammatory mediators and blood brain barrier disruption in fatal brain edema of diabetic ketoacidosis. Brain Res..

[88] Yeung D., Manias J.L., Stewart D.J., Nag S. (2008). Decreased junctional adhesion molecule-A expression during blood-brain barrier breakdown. Acta Neuropathol..

[89] Xu J., Kausalya P.J., Phua D.C., Ali S.M., Hossain Z., Hunziker W. (2008). Early embryonic lethality of mice lacking ZO-2, but Not ZO-3, reveals critical and nonredundant roles for individual zonula occludens proteins in mammalian development. Mol. Cell. Biol..

[90] Fanning A.S., Jameson B.J., Jesaitis L.A., Anderson J.M. (1998). The tight junction protein ZO-1 establishes a link between the transmembrane protein occludin and the actin cytoskeleton. J. Biol. Chem..

[91] Sauer R.S., Kirchner J., Yang S., Hu L., Leinders M., Sommer C., Brack A., Rittner H.L. (2017). Blood-spinal cord barrier breakdown and pericyte deficiency in peripheral neuropathy. Ann. N. Y. Acad. Sci..

[92] Bauer H., Stelzhammer W., Fuchs R., Weiger T.M., Danninger C., Probst G., Krizbai I.A. (1999). Astrocytes and neurons express the tight junction-specific protein occludin in vitro. Exp. Cell Res..

[93] Devaux J., Fykkolodziej B., Gow A. (2010). Claudin Proteins And Neuronal Function. Curr. Top. Membr..

[94] Mandel I., Paperna T., Glass-Marmor L., Volkowich A., Badarny S., Schwartz I., Vardi P., Koren I., Miller A. (2012). Tight junction proteins expression and modulation in immune cells and multiple sclerosis. J. Cell. Mol. Med..

[95] Liu P., Zhang R., Liu D., Wang J., Yuan C., Zhao X., Li Y., Ji X., Chi T., Zou L. (2018). Time-course investigation of blood-brain barrier permeability and tight junction protein changes in a rat model of permanent focal ischemia. J. Physiol. Sci..

[96] Huber J.D., Hau V.S., Borg L., Campos C.R., Egleton R.D., Davis T.P. (2002). Blood-brain barrier tight junctions are altered during a 72-h exposure to (lambda)-carrageenan-induced inflammatory pain. Am. J. Physiol. Heart Circ. Physiol..

[97] Lin J.L., Huang Y.H., Shen Y.C., Huang H.C., Liu P.H. (2010). Ascorbic acid prevents blood-brain barrier disruption and sensory deficit caused by sustained compression of primary somatosensory cortex. J. Cereb. Blood Flow. Metab..

[98] Kaur J., Fahmy L.M., Davoodi-Bojd E., Zhang L., Ding G., Hu J., Zhang Z., Chopp M., Jiang Q. (2021). Waste clearance in the brain. Front. Neuroanat..

[99] Campos-Bedolla P., Walter F.R., Veszelka S., Deli M.A. (2014). Role of the blood-brain barrier in the nutrition of the central nervous system. Arch. Med. Res..

[100] Kaya M., Ahishali B. (2021). Basic physiology of the blood-brain barrier in health and disease: A brief overview. Tissue Barriers.

[101] Doney E., Cadoret A., Dion-Albert L., Lebel M., Menard C. (2022). Inflammation-driven brain and gut barrier dysfunction in stress and mood disorders. Eur. J. Neurosci..

[102] Menard C., Pfau M.L., Hodes G.E., Kana V., Wang V.X., Bouchard S., Takahashi A., Flanigan M.E., Aleyasin H., LeClair K.B. (2017). Social stress induces neurovascular pathology promoting depression. Nat. Neurosci..

[103] Welcome M.O., Mastorakis N.E. (2020). Stress-induced blood brain barrier disruption: Molecular mechanisms and signaling pathways. Pharmacol. Res..

[104] Gryka-Marton M., Grabowska A.D., Szukiewicz D. (2025). Breaking the Barrier: The Role of Proinflammatory Cytokines in BBB Dysfunction. Int. J. Mol. Sci..

[105] Campos C.R., Ocheltree S.M., Hom S., Egleton R.D., Davis T.P. (2008). Nociceptive inhibition prevents inflammatory pain induced changes in the blood-brain barrier. Brain Res..

[106] Antonijevic I., Mousa S.A., Schäfer M., Stein C. (1995). Perineurial defect and peripheral opioid analgesia in inflammation. J. Neurosci..

[107] Beggs S., Liu X.J., Kwan C., Salter M.W. (2010). Peripheral nerve injury and TRV1-expressing primary afferent C-fibers cause opening of the blood-brain barrier. Mol. Pain.

[108] Raja S.N., Carr D.B., Cohen M., Finnerup N.B., Flor H., Gibson S., Keefe F.J., Mogil J.S., Ringkamp M., Sluka K.A. (2020). The revised International Association for the Study of Pain definition of pain: Concepts, challenges, and compromises. Pain.

[109] Cervero F., Merskey H. (1996). What is a noxious stimulus?. Pain Forum.

[110] Reynolds J.A., Henwood M.K., Turtle J.D., Baine R.E., Johnston D.T., Grau J.W. (2019). Brain-Dependent Processes Fuel Pain-Induced Hemorrhage After Spinal Cord Injury. Front. Syst. Neurosci..

[111] Grau J.W., Huang Y.J., Turtle J.D., Strain M.M., Miranda R.C., Garraway S.M., Hook M.A. (2017). When Pain Hurts: Nociceptive Stimulation Induces a State of Maladaptive Plasticity and Impairs Recovery after Spinal Cord Injury. J. Neurotrauma.

[112] Huber J.D., Hau V.S., Mark K.S., Brown R.C., Campos C.R., Davis T.P. (2002). Viability of microvascular endothelial cells to direct exposure of formalin, E-carrageenan, and complete Freund’s adjuvant. Eur. J. Pharmacol..

[113] Bean P.A., Giddings G.A., Tarbet M.M., Johnston D.T., Davis J.A., Lout E.T., Henwood M.K., Borland H.L., Grau J.W. (2025). Pain after traumatic brain injury (TBI) fosters hemorrhage at the site of injury. Exp. Neurol..

[114] Sribnick E.A., Popovich P.G., Hall M.W. (2022). Central nervous system injury-induced immune suppression. Neurosurg. Focus.

[115] Bohatschek M., Werner A., Raivich G. (2001). Systemic LPS injection leads to granulocyte influx into normal and injured brain: Effects of ICAM-1 deficiency. Exp. Neurol..

[116] Martinez-Rizo A.B., Fosado-Rodriguez R., Torres-Romero J.C., Lara-Riegos J.C., Ramirez-Camacho M.A., Arroyo Herrera A.L., Villa de la Torre F.E., Ceballos Gongora E., Arana-Argaez V.E. (2024). Models in vivo and in vitro for the study of acute and chronic inflammatory activity: A comprehensive review. Int. Immunopharmacol..

[117] Seemann S., Zohles F., Lupp A. (2017). Comprehensive comparison of three different animal models for systemic inflammation. J. Biomed. Sci..

[118] Doran S.J., Henry R.J., Shirey K.A., Barrett J.P., Ritzel R.M., Lai W., Blanco J.C., Faden A.I., Vogel S.N., Loane D.J. (2020). Early or Late Bacterial Lung Infection Increases Mortality After Traumatic Brain Injury in Male Mice and Chronically Impairs Monocyte Innate Immune Function. Crit. Care Med..

[119] Vermeij J.D., Aslami H., Fluiter K., Roelofs J.J., van den Bergh W.M., Juffermans N.P., Schultz M.J., Van der Sluijs K., van de Beek D., van Westerloo D.J. (2013). Traumatic brain injury in rats induces lung injury and systemic immune suppression. J. Neurotrauma.

[120] Villalba N., Ma Y., Gahan S.A., Joly-Amado A., Spence S., Yang X., Nash K.R., Yuan S.Y. (2023). Lung infection by Pseudomonas aeruginosa induces neuroinflammation and blood-brain barrier dysfunction in mice. J. Neuroinflamm..

[121] Mei B., Li J., Zuo Z. (2021). Dexmedetomidine attenuates sepsis-associated inflammation and encephalopathy via central alpha2A adrenoceptor. Brain Behav. Immun..

[122] He H.J., Wang Y., Le Y., Duan K.M., Yan X.B., Liao Q., Liao Y., Tong J.B., Terrando N., Ouyang W. (2012). Surgery upregulates high mobility group box-1 and disrupts the blood-brain barrier causing cognitive dysfunction in aged rats. CNS Neurosci. Ther..

[123] Okuma Y., Liu K., Wake H., Zhang J., Maruo T., Date I., Yoshino T., Ohtsuka A., Otani N., Tomura S. (2012). Anti-high mobility group box-1 antibody therapy for traumatic brain injury. Ann. Neurol..

[124] Greene C., Connolly R., Brennan D., Laffan A., O’Keeffe E., Zaporojan L., O’Callaghan J., Thomson B., Connolly E., Argue R. (2024). Blood-brain barrier disruption and sustained systemic inflammation in individuals with long COVID-associated cognitive impairment. Nat. Neurosci..

[125] Anderson T.N., Farrell D.H., Rowell S.E. (2021). Fibrinolysis in Traumatic Brain Injury: Diagnosis, Management, and Clinical Considerations. Semin. Thromb. Hemost..

[126] Moore E.E., Moore H.B., Kornblith L.Z., Neal M.D., Hoffman M., Mutch N.J., Schochl H., Hunt B.J., Sauaia A. (2021). Trauma-induced coagulopathy. Nat. Rev. Dis. Primers.

[127] Fawzy M.S., Toraih E.A. (2015). Data supporting the structural and functional characterization of Thrombin-Activatable Fibrinolysis Inhibitor in breast cancer. Data Brief..

[128] Bardehle S., Rafalski V.A., Akassoglou K. (2015). Breaking boundaries-coagulation and fibrinolysis at the neurovascular interface. Front. Cell. Neurosci..

[129] Patibandla P.K., Tyagi N., Dean W.L., Tyagi S.C., Roberts A.M., Lominadze D. (2009). Fibrinogen induces alterations of endothelial cell tight junction proteins. J. Cell. Physiol..

[130] Lee K.R., Kawai N., Kim S., Sagher O., Hoff J.T. (1997). Mechanisms of edema formation after intracerebral hemorrhage: Effects of thrombin on cerebral blood flow, blood-brain barrier permeability, and cell survival in a rat model. J. Neurosurg..

[131] Hijazi N., Abu Fanne R., Abramovitch R., Yarovoi S., Higazi M., Abdeen S., Basheer M., Maraga E., Cines D.B., Higazi A.A. (2015). Endogenous plasminogen activators mediate progressive intracerebral hemorrhage after traumatic brain injury in mice. Blood.

[132] Fanne R.A., Nassar T., Yarovoi S., Rayan A., Lamensdorf I., Karakoveski M., Vadim P., Jammal M., Cines D.B., Higazi A.A.-R. (2010). Blood-brain barrier permeability and tPA-mediated neurotoxicity. Neuropharmacology.

[133] Whyte C.S., Mutch N.J. (2021). uPA-mediated plasminogen activation is enhanced by polyphosphate. Haematologica.

[134] Schreibelt G., Kooij G., Reijerkerk A., van Doorn R., Gringhuis S.I., van der Pol S., Weksler B.B., Romero I.A., Couraud P.O., Piontek J. (2007). Reactive oxygen species alter brain endothelial tight junction dynamics via RhoA, PI3 kinase, and PKB signaling. FASEB J..

[135] Sumi N., Nishioku T., Takata F., Matsumoto J., Watanabe T., Shuto H., Yamauchi A., Dohgu S., Kataoka Y. (2010). Lipopolysaccharide-activated microglia induce dysfunction of the blood-brain barrier in rat microvascular endothelial cells co-cultured with microglia. Cell. Mol. Neurobiol..

[136] Liu Z., Yao X., Jiang W., Li W., Zhu S., Liao C., Zou L., Ding R., Chen J. (2020). Advanced oxidation protein products induce microglia-mediated neuroinflammation via MAPKs-NF-kappaB signaling pathway and pyroptosis after secondary spinal cord injury. J. Neuroinflamm..

[137] Sabirzhanov B., Li Y., Coll-Miro M., Matyas J.J., He J., Kumar A., Ward N., Yu J., Faden A.I., Wu J. (2019). Inhibition of NOX2 signaling limits pain-related behavior and improves motor function in male mice after spinal cord injury: Participation of IL-10/miR-155 pathways. Brain Behav. Immun..

[138] Jolivel V., Bicker F., Biname F., Ploen R., Keller S., Gollan R., Jurek B., Birkenstock J., Poisa-Beiro L., Bruttger J. (2015). Perivascular microglia promote blood vessel disintegration in the ischemic penumbra. Acta Neuropathol..

[139] Shigemoto-Mogami Y., Hoshikawa K., Sato K. (2018). Activated Microglia Disrupt the Blood-Brain Barrier and Induce Chemokines and Cytokines in a Rat in vitro Model. Front. Cell. Neurosci..

[140] Meng S., Cao H., Huang Y., Shi Z., Li J., Wang Y., Zhang Y., Chen S., Shi H., Gao Y. (2023). ASK1-K716R reduces neuroinflammation and white matter injury via preserving blood-brain barrier integrity after traumatic brain injury. J. Neuroinflamm..

[141] Simard J.M., Tsymbalyuk O., Keledjian K., Ivanov A., Ivanova S., Gerzanich V. (2012). Comparative effects of glibenclamide and riluzole in a rat model of severe cervical spinal cord injury. Exp. Neurol..

[142] Simard J.M., Chen M., Tarasov K.V., Bhatta S., Ivanova S., Melnitchenko L., Tsymbalyuk N., West G.A., Gerzanich V. (2006). Newly expressed SUR1-regulated NC(Ca-ATP) channel mediates cerebral edema after ischemic stroke. Nat. Med..

[143] Martinez-Valverde T., Vidal-Jorge M., Martinez-Saez E., Castro L., Arikan F., Cordero E., Radoi A., Poca M.A., Simard J.M., Sahuquillo J. (2015). Sulfonylurea Receptor 1 in Humans with Post-Traumatic Brain Contusions. J. Neurotrauma.

[144] Jiang B., Li L., Chen Q., Tao Y., Yang L., Zhang B., Zhang J.H., Feng H., Chen Z., Tang J. (2017). Role of Glibenclamide in Brain Injury After Intracerebral Hemorrhage. Transl. Stroke Res..

[145] Popovich P.G., Lemeshow S., Gensel J.C., Tovar C.A. (2012). Independent evaluation of the effects of glibenclamide on reducing progressive hemorrhagic necrosis after cervical spinal cord injury. Exp. Neurol..

[146] Wang X., Chang Y., He Y., Lyu C., Li H., Zhu J., Liu K., Hu Y., Huang K., Pan S. (2020). Glimepiride and glibenclamide have comparable efficacy in treating acute ischemic stroke in mice. Neuropharmacology.

[147] Visse R., Nagase H. (2003). Matrix metalloproteinases and tissue inhibitors of metalloproteinases: Structure, function, and biochemistry. Circ. Res..

[148] Zhang H., Adwanikar H., Werb Z., Noble-Haeusslein L.J. (2010). Matrix metalloproteinases and neurotrauma: Evolving roles in injury and reparative processes. Neuroscientist.

[149] Galis Z.S., Khatri J.J. (2002). Matrix metalloproteinases in vascular remodeling and atherogenesis: The good, the bad, and the ugly. Circ. Res..

[150] Lijnen H.R. (2001). Plasmin and matrix metalloproteinases in vascular remodeling. J. Thromb. Haemost..

[151] Haorah J., Ramirez S.H., Schall K., Smith D., Pandya R., Persidsky Y. (2007). Oxidative stress activates protein tyrosine kinase and matrix metalloproteinases leading to blood-brain barrier dysfunction. J. Neurochem..

[152] de Castro R., Burns C.L., McAdoo D.J., Romanic A.M. (2000). Metalloproteinase increases in the injured rat spinal cord. NeuroReport.

[153] Bauer A.T., Burgers H.F., Rabie T., Marti H.H. (2010). Matrix metalloproteinase-9 mediates hypoxia-induced vascular leakage in the brain via tight junction rearrangement. J. Cereb. Blood Flow Metab..

[154] Mori T., Wang X., Aoki T., Lo E.H. (2002). Downregulation of matrix metalloproteinase-9 and attenuation of edema via inhibition of ERK mitogen activated protein kinase in traumatic brain injury. J. Neurotrauma.

[155] Rosenberg G.A., Yang Y. (2007). Vasogenic edema due to tight junction disruption by matrix metalloproteinases in cerebral ischemia. Neurosurg. Focus.

[156] Ding X.W., Sun X., Shen X.F., Lu Y., Wang J.Q., Sun Z.R., Miao C.H., Chen J.W. (2019). Propofol attenuates TNF-alpha-induced MMP-9 expression in human cerebral microvascular endothelial cells by inhibiting Ca(2+)/CAMK II/ERK/NF-kappaB signaling pathway. Acta Pharmacol. Sin..

[157] Lee T.H., Chen J.L., Tsai M.M., Wu Y.H., Tseng H.C., Cheng L.C., Shanmugam V., Hsieh H.L. (2024). Protective Effects of Sophoraflavanone G by Inhibiting TNF-α-Induced MMP-9-Mediated Events in Brain Microvascular Endothelial Cells. Int. J. Mol. Sci..

[158] Maddahi A., Chen Q., Edvinsson L. (2009). Enhanced cerebrovascular expression of matrix metalloproteinase-9 and tissue inhibitor of metalloproteinase-1 via the MEK/ERK pathway during cerebral ischemia in the rat. BMC Neurosci..

[159] Niego B., Freeman R., Puschmann T.B., Turnley A.M., Medcalf R.L. (2012). t-PA-specific modulation of a human blood-brain barrier model involves plasmin-mediated activation of the Rho kinase pathway in astrocytes. Blood.

[160] Sheth K.N., Albers G.W., Saver J.L., Campbell B.C.V., Molyneaux B.J., Hinson H.E., Cordonnier C., Steiner T., Toyoda K., Wintermark M. (2024). Intravenous glibenclamide for cerebral oedema after large hemispheric stroke (CHARM): A phase 3, double-blind, placebo-controlled, randomised trial. Lancet Neurol..

[161] Kimberly W.T., Battey T.W., Pham L., Wu O., Yoo A.J., Furie K.L., Singhal A.B., Elm J.J., Stern B.J., Sheth K.N. (2014). Glyburide is associated with attenuated vasogenic edema in stroke patients. Neurocrit Care.

[162] Sheth K.N., Kimberly W.T., Elm J.J., Kent T.A., Mandava P., Yoo A.J., Thomalla G., Campbell B., Donnan G.A., Davis S.M. (2014). Pilot study of intravenous glyburide in patients with a large ischemic stroke. Stroke.

[163] Minnema A.J., Mehta A., Boling W.W., Schwab J., Simard J.M., Farhadi H.F. (2019). SCING-Spinal Cord Injury Neuroprotection with Glyburide: A pilot, open-label, multicentre, prospective evaluation of oral glyburide in patients with acute traumatic spinal cord injury in the USA. BMJ Open.

[164] Leigh R., Kern K.C., Wang N.Y., Gottesman R.F., Wright C.B. (2025). Disruption of the Blood-Brain Barrier Predicts Progression of Cerebral Small Vessel Disease White Matter Hyperintensities. Ann. Neurol..

[165] Simpkins A.N., Dias C., Leigh R., National Institutes of Health Natural History of Stroke Investigators (2016). Identification of Reversible Disruption of the Human Blood-Brain Barrier Following Acute Ischemia. Stroke.

[166] Leigh R., Hitomi E., Hutchison R.M., Elkins J. (2021). Post-stroke blood-brain barrier disruption predicts poor outcome in patients enrolled in the ACTION study. J. Neuroimaging.

[167] Montagne A., Nation D.A., Sagare A.P., Barisano G., Sweeney M.D., Chakhoyan A., Pachicano M., Joe E., Nelson A.R., D’Orazio L.M. (2020). *APOE4* leads to blood-brain barrier dysfunction predicting cognitive decline. Nature.

[168] Vos P.E., Lamers K.J.B., Hendriks J.C.M., van Haaren M., Beems T., Zimmerman C., van Geel W., de Reus H., Biert J., Verbeek M.M. (2004). Glial and neuronal proteins in serum predict outcome after severe traumatic brain injury. Neurology.

[169] Foerch C., Singer O.C., Neumann-Haefelin T., du Mesnil de Rochemont R., Steinmetz H., Sitzer M. (2005). Evaluation of serum S100B as a surrogate marker for long-term outcome and infarct volume in acute middle cerebral artery infarction. Arch. Neurol..

[170] Al-Bachari S., Naish J.H., Parker G.J.M., Emsley H.C.A., Parkes L.M. (2020). Blood-Brain Barrier Leakage Is Increased in Parkinson’s Disease. Front. Physiol..

[171] Winkler E.A., Sengillo J.D., Sullivan J.S., Henkel J.S., Appel S.H., Zlokovic B.V. (2013). Blood-spinal cord barrier breakdown and pericyte reductions in amyotrophic lateral sclerosis. Acta Neuropathol..

[172] Li G., Li Z., Sun Y., Bu T., Chen S., Yang L., Li Z., Mao W., Jia Y. (2026). Advances in CNS drug delivery strategies to cross the blood-brain barrier. Chin. Chem. Lett..

[173] Shi X., Wang S., Xiong T., Li R., Zheng W., Chen W., Zhao T., Yang Y., Ying X., Qi W. (2025). Specifically Breaking Through the Injured Blood-Brain Barrier With Tannic Acid-Based Nanomedicine for Ischemic Stroke Ischemia Reperfusion Treatment. Exploration.

[174] Krolak T., Chan K.Y., Kaplan L., Huang Q., Wu J., Zheng Q., Kozareva V., Beddow T., Tobey I.G., Pacouret S. (2022). A High-Efficiency AAV for Endothelial Cell Transduction Throughout the Central Nervous System. Nat. Cardiovasc. Res..

[175] Martin M., Vermeiren S., Bostaille N., Eubelen M., Spitzer D., Vermeersch M., Profaci C.P., Pozuelo E., Toussay X., Raman-Nair J. (2022). Engineered Wnt ligands enable blood-brain barrier repair in neurological disorders. Science.

[176] Sun Z.W., Wang X., Zhao Y., Sun Z.X., Wu Y.H., Hu H., Zhang L., Wang S.D., Li F., Wei A.J. (2024). Blood-brain barrier dysfunction mediated by the EZH2-Claudin-5 axis drives stress-induced TNF-alpha infiltration and depression-like behaviors. Brain Behav. Immun..

[177] Human Protein Atlas. https://www.proteinatlas.org/.

[178] Sjostedt E., Zhong W., Fagerberg L., Karlsson M., Mitsios N., Adori C., Oksvold P., Edfors F., Limiszewska A., Hikmet F. (2020). An atlas of the protein-coding genes in the human, pig, and mouse brain. Science.

[179] Davison D.L., Terek M., Chawla L.S. (2012). Neurogenic pulmonary edema. Crit. Care.

[180] Jogia T., Ruitenberg M.J. (2020). Traumatic Spinal Cord Injury and the Gut Microbiota: Current Insights and Future Challenges. Front. Immunol..

[181] Stanley D., Mason L.J., Mackin K.E., Srikhanta Y.N., Lyras D., Prakash M.D., Nurgali K., Venegas A., Hill M.D., Moore R.J. (2016). Translocation and dissemination of commensal bacteria in post-stroke infection. Nat. Med..

[182] G.B.D. Diseases and Injuries Collaborators (2020). Global burden of 369 diseases and injuries in 204 countries and territories, 1990–2019: A systematic analysis for the Global Burden of Disease Study 2019. Lancet.

[183] Guan B., Anderson D.B., Chen L., Feng S., Zhou H. (2023). Global, regional and national burden of traumatic brain injury and spinal cord injury, 1990–2019: A systematic analysis for the Global Burden of Disease Study 2019. BMJ Open.

[184] Detloff M.R., Fisher L.C., McGaughy V., Longbrake E.E., Popovich P.G., Basso D.M. (2008). Remote activation of microglia and pro-inflammatory cytokines predict the onset and severity of below-level neuropathic pain after spinal cord injury in rats. Exp. Neurol..

[185] Walters E.T. (2014). Neuroinflammatory contributions to pain after SCI: Roles for central glial mechanisms and nociceptor-mediated host defense. Exp. Neurol..

